# Effects of Resistance Training on Measures of Muscular Strength in People with Parkinson’s Disease: A Systematic Review and Meta-Analysis

**DOI:** 10.1371/journal.pone.0132135

**Published:** 2015-07-06

**Authors:** Luisa Roeder, Joseph T. Costello, Simon S. Smith, Ian B. Stewart, Graham K. Kerr

**Affiliations:** 1 Institute of Health and Biomedical Innovation, Queensland University of Technology, Brisbane, Australia; 2 Movement Neuroscience Program, Institute of Health and Biomedical Innovation, Queensland University of Technology, Brisbane, Australia; 3 Injury Prevention Program, Institute of Health and Biomedical Innovation, Queensland University of Technology, Brisbane, Australia; 4 School of Exercise and Nutrition Sciences, Queensland University of Technology, Brisbane, QLD, Australia; 5 Extreme Environments Laboratory (EEL), Department of Sport and Exercise Science, Spinnaker Building, Cambridge Road, University of Portsmouth, Portsmouth, PO1 2ER, United Kingdom; 6 CARRS-Q, School of Psychology and Counselling, Queensland University of Technology, Brisbane, Australia; Cardiff University, UNITED KINGDOM

## Abstract

**Objective:**

The aim of this systematic review and meta-analysis was to determine the overall effect of resistance training (RT) on measures of muscular strength in people with Parkinson’s disease (PD).

**Methods:**

Controlled trials with parallel-group-design were identified from computerized literature searching and citation tracking performed until August 2014. Two reviewers independently screened for eligibility and assessed the quality of the studies using the Cochrane risk-of-bias-tool. For each study, mean differences (MD) or standardized mean differences (SMD) and 95% confidence intervals (CI) were calculated for continuous outcomes based on between-group comparisons using post-intervention data. Subgroup analysis was conducted based on differences in study design.

**Results:**

Nine studies met the inclusion criteria; all had a moderate to high risk of bias. Pooled data showed that knee extension, knee flexion and leg press strength were significantly greater in PD patients who undertook RT compared to control groups with or without interventions. Subgroups were: RT vs. control-without-intervention, RT vs. control-with-intervention, RT-with-other-form-of-exercise vs. control-without-intervention, RT-with-other-form-of-exercise vs. control-with-intervention. Pooled subgroup analysis showed that RT combined with aerobic/balance/stretching exercise resulted in significantly greater knee extension, knee flexion and leg press strength compared with no-intervention. Compared to treadmill or balance exercise it resulted in greater knee flexion, but not knee extension or leg press strength. RT alone resulted in greater knee extension and flexion strength compared to stretching, but not in greater leg press strength compared to no-intervention.

**Discussion:**

Overall, the current evidence suggests that exercise interventions that contain RT may be effective in improving muscular strength in people with PD compared with no exercise. However, depending on muscle group and/or training dose, RT may not be superior to other exercise types. Interventions which combine RT with other exercise may be most effective. Findings should be interpreted with caution due to the relatively high risk of bias of most studies.

## Introduction

Parkinson’s disease (PD) is the second most common neurodegenerative disorder after Alzheimer’s disease [1] and affects approximately six million people worldwide [2]. PD is more prevalent in older age groups with a rapid increase of cases after the age of 60 [3,4]. The incidence rate adjusted for age is estimated to be 9.7 to 13.8 cases per 100,000 people per year [5]. It is expected that these numbers will increase further in the next few decades due to an aging population [6].

PD is a chronic and progressive disorder that is thought to be caused by death of dopaminergic neurons in the substantia nigra of the basal ganglia [7]. There is emerging evidence that other non-dopaminergic structures are also involved [8]. PD includes motor and non-motor symptoms [1,9]. Non-motor symptoms include a decline in cognitive function, psychiatric problems such as depression and anxiety, and autonomic, sleep, and sensory disturbances [10]. Common motor symptoms are tremor, bradykinesia (slowness of movement), rigidity, postural instability and a stooped posture, gait difficulties including freezing of gait (inability to initiate movement), and muscle weakness [1,2,11]. These movement difficulties lead to decreased activity levels in people with PD which, in turn, further impairs strength and physical functioning. Impaired muscular strength may be a primary symptom inherent in PD [12], but this remains controversial [13]. Impaired strength may be of central origin [14], as the ability to activate motor neurons of the active muscle might be impaired due to deficient cortical drive to the muscle [15]. Moreover, muscle weakness may contribute to postural instability and gait difficulties [16,17] and has been identified as a secondary cause for bradykinesia in PD [18].

Available treatment options for PD include pharmacological therapy (dopamine replacement), brain surgery (deep brain stimulation (DBS)) and exercise [1,2]. While there is no cure for PD, these therapies aim to provide symptom relief [7]. Medication and surgery are effective in alleviating the cardinal symptoms (tremor, bradykinesia, rigidity). However, pharmacological therapy only insufficiently improves balance- and gait-disorders and can cause disabling side-effects that become more prominent as the disease progresses [19]. DBS can provide improvements in balance and gait to some extent but its effectiveness is dependent on the stimulation site in the brain and medication co-effects, and decreases over time [20].

Exercise has been shown to be beneficial for people with PD [2,21]. In particular, resistance training (RT) has been shown to improve strength, and some measures of physical function and mobility in PD patients [22–25]. Moreover, it has been demonstrated that increases in muscular strength in response to RT are accompanied by cellular adaptative mechanisms like myofiber hypertrophy in people with PD [26]. RT might also have a neuro-protective effect and slow down disease progression [15,27].Yet, evidence for these beneficial effects arises from a broad variety of RT and the overall effect of RT on measures of muscular strength is unknown. Currently, there are few evidence-based guidelines for RT for people with PD [28]. Therefore, this systematic review aims to (1) collate studies that utilized RT to improve muscular strength in people with PD and update previous reviews, (2) determine the overall effect of RT on measures of muscular strength in people with PD, and (3) identify effective RT interventions to increase strength in people with PD in order to provide evidence-based guidelines for health professionals prescribing RT to PD patients.

## Methods

### Literature Search Strategy

The literature search was performed in MEDLINE, the Cochrane library, CINAHL, EMBASE, and SPORTDiscus. MeSH or keywords and matching synonyms were combined, including Parkinson’s disease, resistance training, and controlled clinical trials. Subject headings were modified for use in the other databases. A copy of the full search strategy in each database can be found in the supporting information (S2 Appendix). Each database was searched from their earliest available record up to 2014 August 15^th^. Reference lists of all relevant articles were also examined for identification of further eligible studies.

### Inclusion and Exclusion Criteria

RT was defined as a form of strength training that is designed to improve components of muscular fitness including strength, power and endurance. It involves the activation of motor units against an external resistance which may be applied to whole body movements or isolated muscle groups. A range of equipment can be used to apply external resistance, for instance bodyweight, free weights, machines with additional weights, elastic bands or water pressure. A RT program is designed by adjusting acute training variables such as the choice of exercises, the order of exercises, frequency of exercise sessions, number of sets and repetitions, intensity levels and rest periods [29,30].

Studies meeting the following criteria were considered for the review: 1) participants of the study had to have PD (any age, any concurrent drug therapy, any disease duration or severity); 2) at least one group of the study must have undergone a RT intervention (> 2 weeks of exercise in order to see a physiological strength change not a neurological improvement in muscle fiber recruitment [31]); 3) at least one outcome measure of muscle strength was reported; 4) the study design was a parallel group design of some sort (i.e. it included at least two arms with an intervention group that performed RT and a control group which did not receive treatment other than standard medical practice or underwent another type of intervention that did not include strengthening exercises). RT studies that did not report acute training variables in a detailed manner and studies that applied strengthening exercises to both/all groups (e.g. comparing two different types of resistance training) were not considered. Only fully peer-reviewed articles with full text available in English were considered.

### Selection of Studies

The initial search was undertaken by one researcher (LR). Titles and abstracts of publications obtained by the search strategy were screened and only those that were obviously outside the scope of the review were removed. We were over-inclusive at this stage and received the full text for any papers that potentially met the review inclusion criteria. Following title/abstract screening, two authors (LR, IBS) independently selected trials for inclusion; based on the information within the full reports, eligible trials were included in the review. All trials classified as eligible by either author were retrieved. Disagreement between the authors was resolved by consensus, or third-party adjudication (JTC, GKK).

### Data Extraction and Management

Data were extracted by two review authors using a customized form (LR, JTC). This was used to extract relevant data on methodological design, eligibility criteria, interventions (including detailed characteristics of the training protocols), participants, comparisons and outcome measures. There was no blinding to study author, institution or journal at this stage.

### Risk of Bias

For all included studies, methodological quality was assessed by two authors independently, using the Cochrane risk-of-bias tool [32]. Each study was graded for the following domains: sequence generation, allocation concealment, blinding (participants & personnel, outcome assessors), incomplete outcome data and selective reporting. For each study, the domains were described as reported in the published study report (or if appropriate based on information from related protocols, or published comments) and judged by the review authors as to their risk of bias according to Section 8.5 of the Cochrane handbook [33]. They were assigned a rating of ‘low’ if criteria for a low risk of bias were met or ‘high’ if criteria for a high risk of bias were met. The risk of bias was deemed ‘unclear’ for a domain if insufficient detail of what happened in the study was reported, or if what happened in the study was known, but the risk of bias was unknown. Disagreements between authors regarding the risk of bias for domains were resolved by consensus.

### Measures of Treatment Effect

For each study, mean differences (MD) or standardized mean differences (SMD) and 95% confidence intervals (CIs) were calculated for continuous outcomes using the Cochrane Collaboration’s software RevMan version 5.2 [34]. As advised in chapter 7.7.3.1 and 9.4.5.2 of the Cochrane handbook [33] treatment effect estimates (MD, SMD) were based on between-group comparisons using post-intervention data (comparison of final values across groups). When values were missing from continuous data, the authors of the article were contacted. There was one case where standard deviation values were missing [35] which were retrieved after correspondence with the authors. In the event that there was no evidence of heterogeneity of effect (P>0.1), a fixed-effect model was used for meta-analysis. In cases where there was evidence of statistical heterogeneity, we checked the results using a random-effects mode.

### Assessment of Heterogeneity

Assessment of heterogeneity between comparable trials was evaluated visually with the use of forest plots, as well as Chi² tests and I² statistics, as outlined in chapter 9.5 of the Cochrane handbook [33]. The level of significance for the Chi² test was set at P = 0.1: a P value for Chi² < 0.1 was considered to indicate statistically significant heterogeneity between studies. Values of I² were interpreted as follows: 0%to 40% might not be important; 30% to 60% may represent moderate heterogeneity; 50% to 90% may represent substantial heterogeneity; and 75% to 100% may represent considerable heterogeneity.

### Subgroup Analysis

Differences in study designs were considered for subgroup analysis. The studies were grouped into four categories as depicted in Table 1: 1) RT vs. control-without-intervention; 2) RT vs. control-with-intervention; 3) RT with other form of exercise vs. control-without-intervention; 4) RT with other form of exercise vs. control-with-intervention. The subgroup analysis was ad hoc and determined by the available literature. The authors decided on the four categories as they were logical and defined the majority of the included studies.

**Table 1:** Study Characteristics.

Study	Participants and Groups Number, sex (f;m), age (yrs), disease details (HY, PD dura)	Resistance Training Program (duration, frequency, exercises, volume, intensity, progression)	Outcome Measures of Strength	Results, Findings (WGC: BL vs. post; BGC: post RT group vs. post other group)		
***Resistance training vs*. *control-without-intervention***						
Bloomer et al. (2008) *PGS* [35]	1) RT—8 PD (4;4), 61 ± 2, HY n/a (1–2), PD dura n/a	8 wks, 2 days/wk	BILATERAL 1 RM	Strength leg press		
	2) Con PD—8 PD (4;4), 57 ± 3, HY n/a (1–2), PD dura n/a	Machine leg press, knee flx, calf press	machine-based leg press (kg)		WGC	BGC
		3 x 5–8, each set to a momentary failure	Tested/trained ON	RT	↑	→
		5–10% load increase when performance of 3 x 8 successful		Con PD	→	→
Schilling et al. (2010) *PGS* [41]	1) RT PD—8 PD (3;5), 61.3 ± 8.6, HY 2.1 (1–2.5), PD dura n/a, UPDRS total 19.1±7.0	8wks, 2 days/wk	BILATERAL 1 RM	Strength leg press		
	2) Con PD—7 PD (3;4), 57.0 ± 7.1, HY 1.9 (1–2.5), PD dura n/a, UPDRS total 23.3 ± 18.0	Machine leg press, knee flx, calf press	machine-based leg press (kg/kg)		WGC	BGC
		3 x 5–8: initial load established via trial and error, requirement: subject is able to perform 2 x 8 + 1 x 5–8; Conc phase: fast, ecc: slow	Tested ON	RT	↑	→
		load increase of 5–10% when 3 x 8 achieved		Con PD	→	→
***Resistance training vs*. *control-with-intervention***						
Li et al. (2012) *RCT* [40]	1) RT—65 PD (27;38), 69 ± 8, HY (1–4), PD dura 8 ± 9, UPDRS motor 15.32±6.04	24 wks, 2days/wk, 60 min./session	BILATERAL ISOKINETIC DYNAMOMETER	Strength knee ext/flx		
	2) Stretch—65 PD (26;39), 69 ± 9, HY (1–4), PD dura 6 ± 5, UPDRS motor 15.06±6.17	Forward/side steps, squats, forward/side lunges, heel/toe raises with weighted vests & ankle weights	Peak torque (Nm)		WGC	BGC
	3) Tai Chi—65 PD (20;45), 68 ± 9, HY (1–4), PD dura 8 ± 9, UPDRS motor 15.28±5.59	wk 1–9: 1–3 x 10–15 body weight, wk 10–14: 1–3 x 10–15 weights 1–2% of body weight, wk 15–19: 1–3 x 10–15 weights 2–4% of body weight, wk 20–24: 1–3 x 10–15 weights 3–5% of body weight	1. knee ext at 60°.sec^-1^, 2. knee flx at 60°.sec^-1^	RT	↑	↑ (vs. Stretch)
		(increase of resistance every 5^th^ week)	Tested ON	Stretch	→	→ (vs. RT)
				Tai Chi	↑	↑ (vs. Stretch)
***Resistance training with other form of exercise vs*. *control-without-intervention***						
Bridge-water et al. (1997) *PGS* [37]	1) Exc―13 PD (4;9), 67.3 ± 3.9, HY 2.1 (1–3), PD dura 4 ±2.4	12 wks, 2 days/wk	MAX. ISOMETRIC DYNAMOMETER	Strength trunk flx/ext/rotation		
	2) Con―13PD (6;7), 65.9 ± 10.2, HY 2.0 (1–3), PD dura 4 ± 3.2	1x10: 4 abdominal exercises supine	Max & avg torque (Nm)		WGC	BGC
		1x10 of 7s isometric contractions: upper back prone, lower back prone, on-all-fours exercises (as the subjects ability improved they got more advanced exercises, but overall bodyweight only)	1. trunk flx (from neutral), 2. trunk ext (from 10° flx), 3. right trunk rotation (from neutral), 4. left trunk rotation (from neutral)	Exc	↑	↑
		Aerobic training	2x6sec contractions	Con	→	→
Toole et al. (2000) *PGS* [43]	1) RT + Bal—4 PD (2;2), 73, HY n/a (1–3), PD dura n/a	10 wks, 3 days/wk, 60 min./session	UNILATERAL ISOKINETIC DYNAMOMETER	Strength knee ext/flx		
	2) Con—3 PD (1;2), 71, HY n/a (1–3), PD dura n/a	Machine knee flx/ext, theraband ankle inversion, Balance exercises	Peak torque (ft-lb) right leg		WGC	BGC
		3 x 10 at 60% 4 RM, 6s contraction (2conc-4ecc), weekly readjusted	1. knee ext at 90°.sec^-1^ and 180°.sec^-1^, 2. knee flx at 90°.sec^-1^ and 180°.sec^-1^, 3. ankle inversion at 120°.sec^-1^	RT+Bal	→	→
			Tested ON	Con	↓	→
Allen et al. (2010) *RCT* [36]	1) Exc―24 PD (11;13), 66±10, HY n/a, PD dura 7±5, UPDRS motor 29 ±10	6 months, 3 days/wk (1x per month supervised group session, remaining sessions at home), 40–60 min./session	UNILATERAL STRAIN GAUGE	Strength knee ext		
	2) Con―24PD (11;13), 68±7, HY n/a, PD dura 9±6, UPDRS motor 30 ± 15	Standing up and sitting down, heel raises in standing, half squats, forward or lateral step-ups onto a block	(kg), knee ext, weaker leg, stronger leg, average		WGC	BGC
		wk 1: 2 x 10 body weight or weighted vests up to 2% of body weight, 3 exercises only; from wk 1 onwards: 10–15 reps, more exercises		Exc	→	→
		progression (load increase) individually tailored aimed to reach RPE = 15 (“hard”) on Borg Scale, readjusted every 2–4 wks; Balance exercises		Con	→	→
DiFran-cisco-Donoghue et al. (2012) *PGS* [38]	1) Exc―9PD (2;7), 68 ±7, HY 2, PD dura 8 ± 5	6 wks, 2 days/wk, 40 min./session	1RM	Strength knee ext/flx/leg press		
	2) Exc+Vit―9PD (5;5), 67 ±6, HY 2, PD dura 7 ± 4	20 min. aerobic training (treadmill), 20 min. machine-based resistance training: knee ext/flx, leg press, arm curl, chest fly	in lb		WGC	BGC
	3) Vit―9PD (4;5), 69 ±7, HY 2, PD dura 9 ± 6	2x8-15 at 50–80% 1RM, 30s rest	1. knee ext, 2. knee flx, 3. leg press	Exc	↑	↑ (vs. Con)
	4) Con―9PD (6;3), 68 ±8, HY 2, PD dura 9 ± 6	5lb load increase when 1x15 successfully performed	Tested ON	Exc+Vit	↑	↑ (vs. Con)
				Vit	→	→ (vs. Con)
				Con	→	→ (vs. Exc)
***Resistance training with other form of exercise vs*. *control-with- intervention***						
Hirsch et al. (2003) *RCT* [39]	1) RT+Bal―6 PD, 70.8 ± 2.8, HY 1.8 ± 0.3, PD dura n/a	10 wks, 3 days/wk, 15 min./session	BILATERAL 4 RM,	Strength knee ext/flx/plantarflx		
	2) Bal―9 PD, 75.7 ± 1.8, HY 1.9 ± 0.6, PD dura n/a	Machine knee flx/ext, plantarflx, Balance exercises	machine-based (kg)		WGC	BGC
		1 x 12 at 60% 4RM wk 1–2, 1 x 12 at 80% 4RM wk 3–10, 6-9s contraction, 2 min. rest between exercises, fortnightly readjusted	1. knee ext (seated, from 90° of knee flx to full knee ext), 2. knee flx (seated, from 170° of knee ext to 90° of knee flx), 3. plantarflx (seated, from 90° of ankle flx to max plantarflx)	RT+Bal	↑	↑
			Tested ON	Bal	→	→
Shulman et al. (2013) *RCT* [42]	1) RT―22PD (4;18), 65.3 ± 11.3, HY ON 2.2 (2–3), PD dura 6.3 ± 4.0, UPDRS total 48.2 ± 15.5; UPDRS motor 34.5 ± 10.7	3 months, 3 days/wk	UNILATERAL 1 RM	Strength leg press/knee ext		
	2) HIT―23PD (7;16), 66.1 ± 9.7, HY ON 2.2 (2–3), PD dura 5.9 ± 3.9, UPDRS total 45.2 ± 12.2; UPDRS motor 30.3 ± 9.8	Machine leg press, knee ext, knee flx	machine-based		WGC	BGC
	3) LIT―22PD (6;16), 65.8 ± 11.5, HY ON 2.2 (2–3), PD dura 6.3 ± 3.5, UPDRS total 46.6 ± 12.6; UPDRS motor 31.6 ± 9.2	2 x 10 at? % 1RM, load increased as tolerated	1. leg press (lb), 2. knee ext (lb)	RT	↑	→
		Stretching: trunk rotation, hip abduction, and stretches of hamstrings, quadriceps, calves, and ankles (1 x 10)		HIT	→	→
				LIT	→	→

**↑**increase; **→** no changes; **1RM** = one-repetition maximum; **ab** = abdominal; **avg** = average; **Bal** = balance training; **BGC** = between-group comparison; **BL** = baseline; **Con** = control group; **conc** = concentric; **ecc** = eccentric; **exc** = exercise; **ext** = extension; **f** = female; **flx** = flexion; **HIT** = high-intensity treadmill training; **HY** = mean Hoehn & Yahr score ± SD (range); **lat** = latissimus dorsi; **LIT** = low-intensity treadmill training; **m** = male; **max.** = maximal; **OFF** = patients were on an overnight withdrawal of medication; **ON** = patients had taken parkinsonian medication; **PD** = Parkinson’s disease; **PD dura** = mean duration of PD in years ± SD (range) since diagnosis; **PGS** = parallel group study; **post** = post intervention; **RCT** = randomized controlled trial; **RPE** = rating of perceived exertion; **RT** = resistance training; **sc** = standard care; **TMW** = 10 m walk test; **TUG** = timed up and go; **WGC** = within-group comparison; **wk** = week (duration); **Vit** = vitamin supplementation, **yrs** = mean age ± SD (range).

## Results

**Fig 1:**
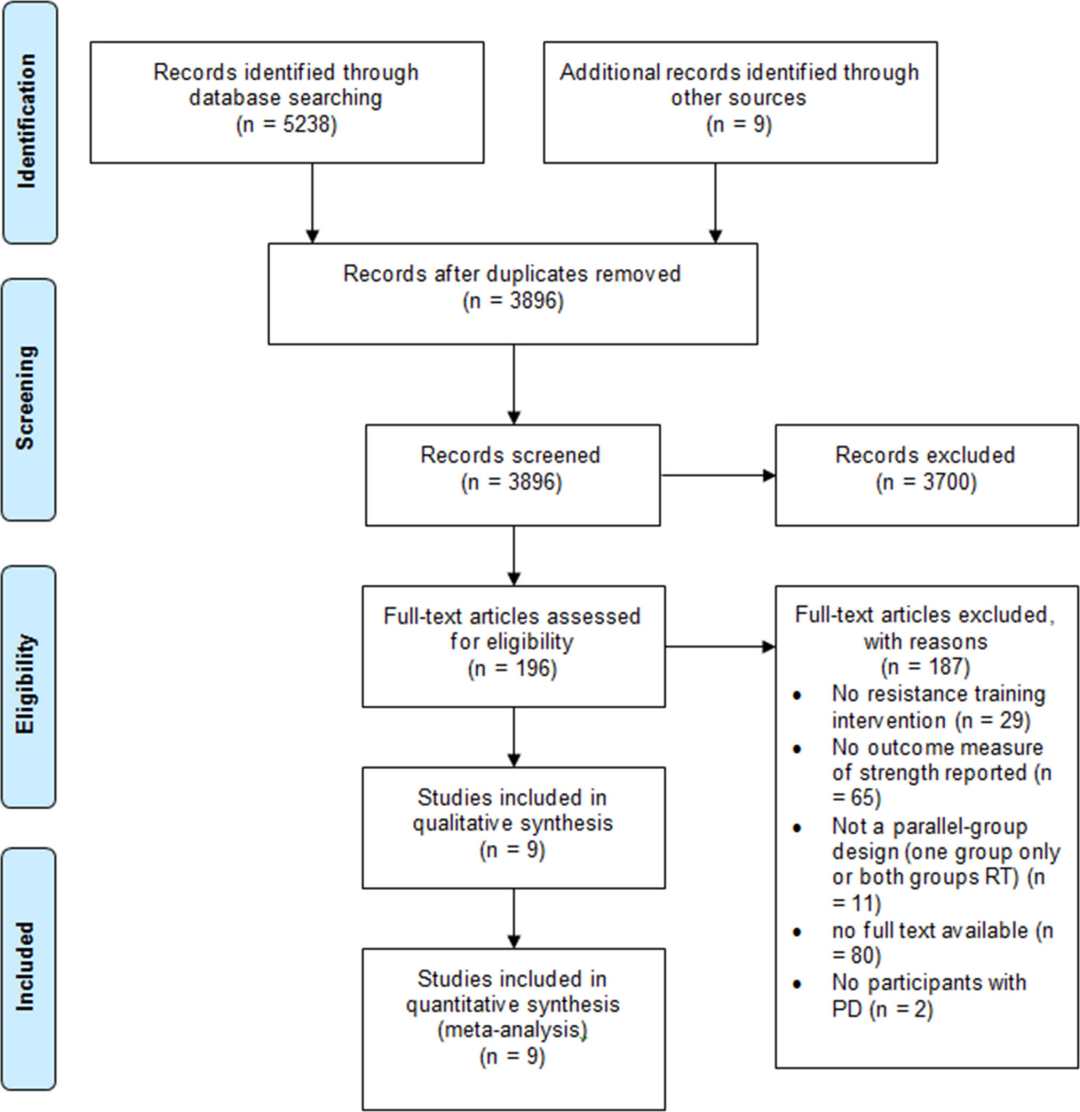
Summary of search and selection process based on included and excluded studies.

Fig 1 summarizes the search and selection process based on included and excluded studies.

### Included studies

Characteristics of included studies are summarized in Table 1. There were nine eligible studies [35–43]. Of the nine included studies, four were randomized controlled trials (RCTs) [36,39,40,42], one study was match-randomized according to disease severity and gender [35], one study was gender-match-randomized [41] and three studies incorporated an intervention group and a control group (parallel group design) without further details on their study design [37,38,43]. Two studies [35,41] compared RT alone with a control group that received standard medical practice; one study (RCT) [40] compared RT with another intervention (Tai Chi or Stretching); four studies [36–38,43] including one RCT [36] compared an intervention that comprised RT combined with another form of exercise (aerobic or balance training) with a control group that received standard medical practice; two studies [39,42] (both RCTs) compared an intervention that comprised RT combined with another form of exercise (balance training or stretching) with another intervention that did not contain any strengthening component.

### Study Cohort

The nine included studies comprised a total of 425 participants with PD. Overall, 168 participants followed a RT regime, 257 were part of a control group (standard medical practice) or another intervention (stretching, balance, treadmill training). The sample size was 47 ± 58 [mean ± SD]. Of all participants 254 were male (59.8%), 156 participants (36.7%) were female and the sex of 15 (3.5%) was not reported. All but one study [35] reported the age of their participants; overall, the mean age of participants was 67.7 ± 8.8 years.

PD severity was described using the Hoehn and Yahr scale (H&Y) [44] in all but one study [36]; however, some studies reported means and standard deviations/errors while others reported the range only. The H&Y scale gives an overall estimate of symptom severity from stage 1 (little signs of disease, unilaterally) to 5 (severe disability, wheelchair bound). The majority of studies included participants with low to moderate disease severity according to the H&Y scale (for details see Table 1). The Unified Parkinson’s Disease Rating scale (UPDRS) [45], as another measure of PD status, was described in three studies [36,41,42] (see Table 1 for details). The PD duration was reported in five studies [36–38,40,42] and the mean was 7.1 ± 1.7 years.

### Training Dose

Reporting of acute training variables across all studies was highly variable. Details about training duration, frequency, volume, intensity, progression, resting periods, movement velocity, which muscle groups were targeted, which equipment was used and about supervision arrangements are collated in Table 1. It is important to note, though, that not all studies provided information on all of those training variables.

In summary, the majority of studies targeted the lower limbs in their RT [35,36,39–43], particularly the knee extensors and flexors, hip extensors and plantarflexors and conducted machine-based training [35,38,39,41–43]. Intervention durations ranged from six weeks [38] to six months [36,40]. Exercise frequency was either two [35,37,38,40,41] or three days per week [36,39,42,43]. Training volume ranged from one to three sets with five to 15 repetitions with or without increasing volume over the course of the intervention. Only two studies reported the duration of rest periods between sets or exercises (30s [38] and 120s [39]). Three studies provided some details regarding movement velocity during each repetition [39,41,43].

Intensity levels were specified in only three studies [38,39,43]. Five studies described intensity levels in a more indirect way, such as maximal effort to volitional fatigue [35,41], aim to reach a rating of perceived exertion (RPE) of 15 (‘hard’) on the Borg Scale [36], or percent of bodyweight used as resistance [37,40]. One study did not report any information on the intensity of the exercise [42]. All but one study [37] conducted *progressive* resistance training. How progression was implemented was highly heterogeneous in the included studies (Table 1).

Six interventions were supervised [35,37,39–42] and one study was a home-based intervention which included a supervised group session once a month [36].

### Details of Outcome

All nine studies recorded muscle strength and all but one study [37] assessed lower limb muscle strength. Knee extensor strength was most commonly reported [36,38–40,42,43]. Four studies [38–40,43] measured knee flexor strength and four studies [35,38,41,42] assessed leg press strength. Two studies recorded ankle muscle strength with plantarflexion [39] and inversion [43] and one study reported strength measures of the trunk (flexion, extension, rotation) [37].

Nonetheless, the ways in which strength was measured was heterogeneous (see Table 1). Some studies conducted strength testing via isokinetic or isometric dynamometry [37,40,43] with different specifications, other studies conducted repetition-maximum (RM) strength tests [35,38,39,41,42] with different testing protocols or used a strain gauge [36]. Units of the strength measurements varied across studies (kg, lb, kg/kg, Nm, ft-lb) and so did reporting of the outcomes (e.g. whether peak torque was reported of mean torque) and of testing protocols (e.g. seat and leg/body position, joint angles, unilateral or bilateral testing, number of sets).

### Follow-Up

All studies [35–43] recorded outcomes before and immediately after (pre-post) the RT intervention. Three studies undertook additional outcome assessments at four weeks [37,39] or three months [40] after completion of the intervention. Additionally, one study that ran over six months also undertook outcome assessments midway through the study (at three months) [40].

### Risk of Bias

**Fig 2:**
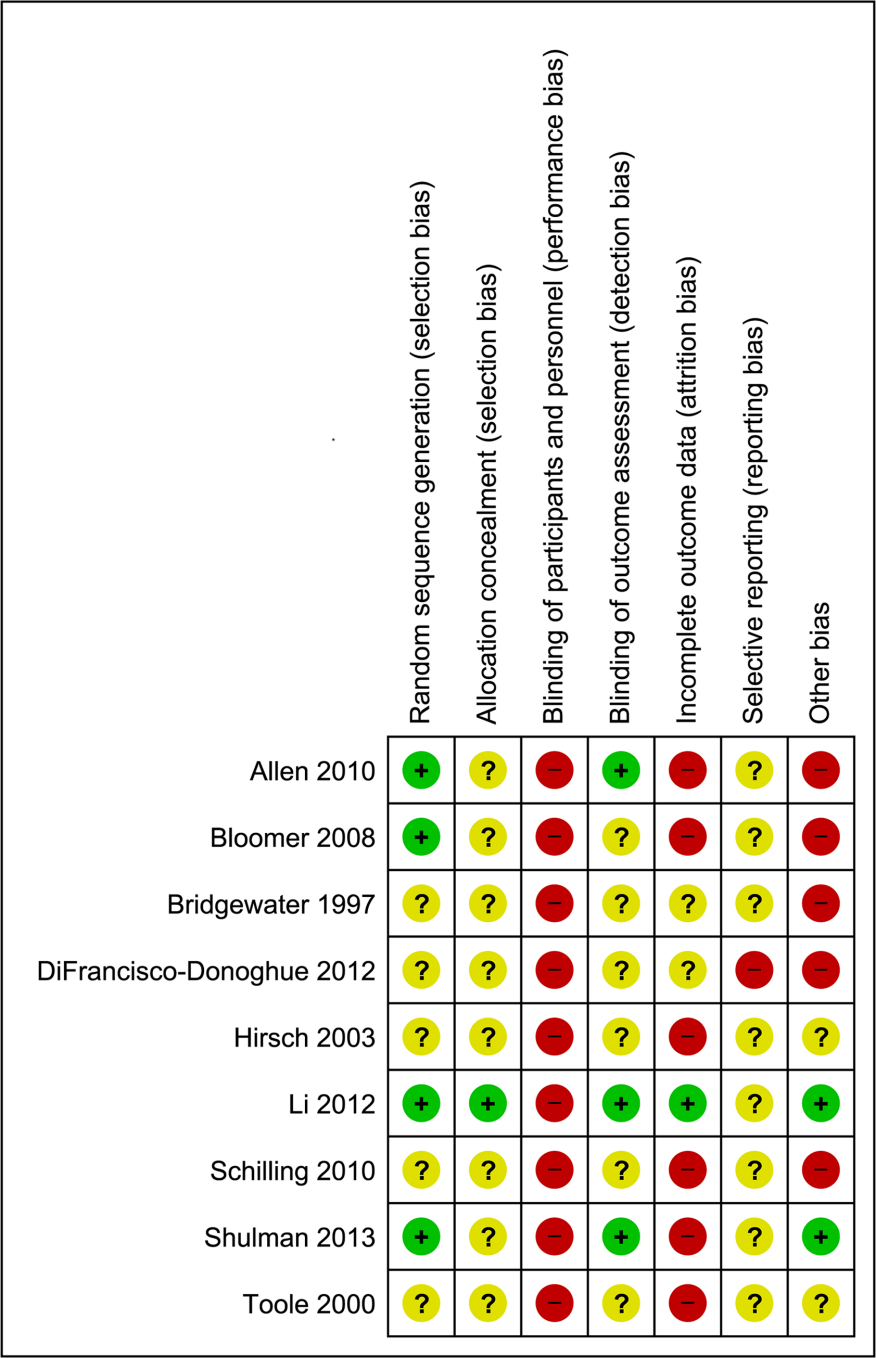
Risk of bias summary: review authors' judgements about each risk of bias item for each included study.

**Fig 3:**
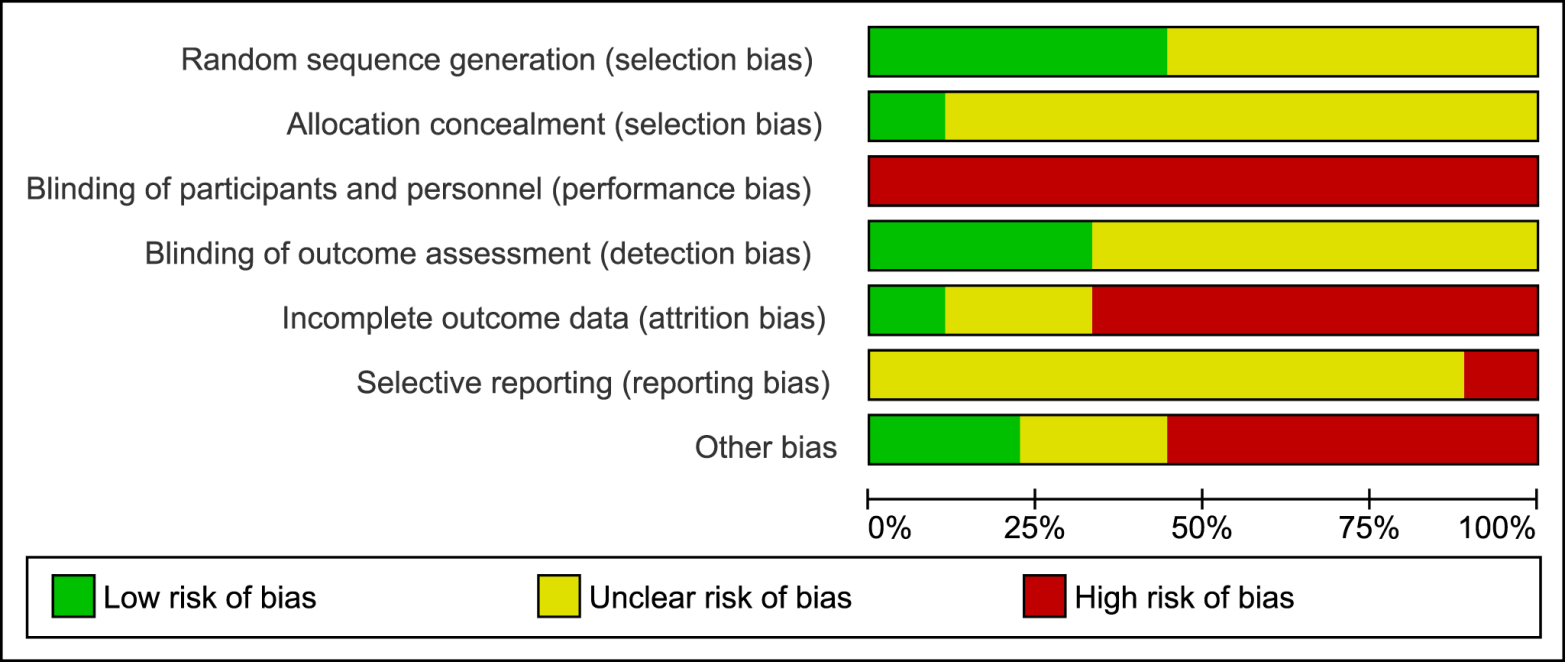
Risk of bias graph: review authors' judgements about each risk of bias item presented as percentages across all included studies.

There was a moderate to high risk of bias across all studies (Figs 2 and 3). Due to the nature of the intervention none of the studies utilized blinding of participants or personnel administering the exercises. Blinding of outcome assessors was reported in three studies [36,40,42]. There was a high risk of attrition bias across all studies; only one study [40] provided appropriate information relating to dropouts, exclusions, missing data and approach to analysis (intention-to-treat). Likewise, only one study [38] made any reference to a published protocol. Despite all studies stating that some form of randomization was employed, only four studies [35,36,40,42] provided adequate details on sequence generation and only one study [40] adequately reported allocation concealment.

### Muscle Strength

#### Muscle Strength: Knee Extension

**Fig 4:**
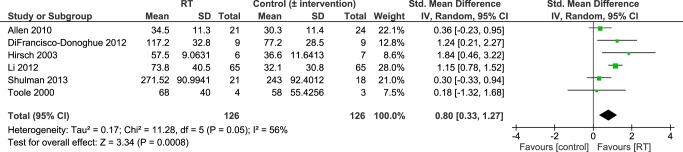
Primary analysis forest plot of comparison: RT vs. control-without/with-intervention, using post-intervention values, outcome: knee extension strength. CI = confidence interval; IV = inverse variance; SMD = standardized mean difference.

Six studies [36,38–40,42,43] reported knee extensor strength as an outcome. Overall, pooled data revealed significantly higher knee extensor strength in people who had undergone an intervention that contained RT compared to controls-without-intervention (standard medical practice) or controls-with-intervention (i.e. people who had undergone another intervention) (SMD 0.80 [95% CI 0.33, 1.27]; Fig 4). Because there was a significant level of heterogeneity between studies (P = 0.05; I^2^ = 56%) sensitivity analysis using a random effects model was performed.

**Fig 5:**
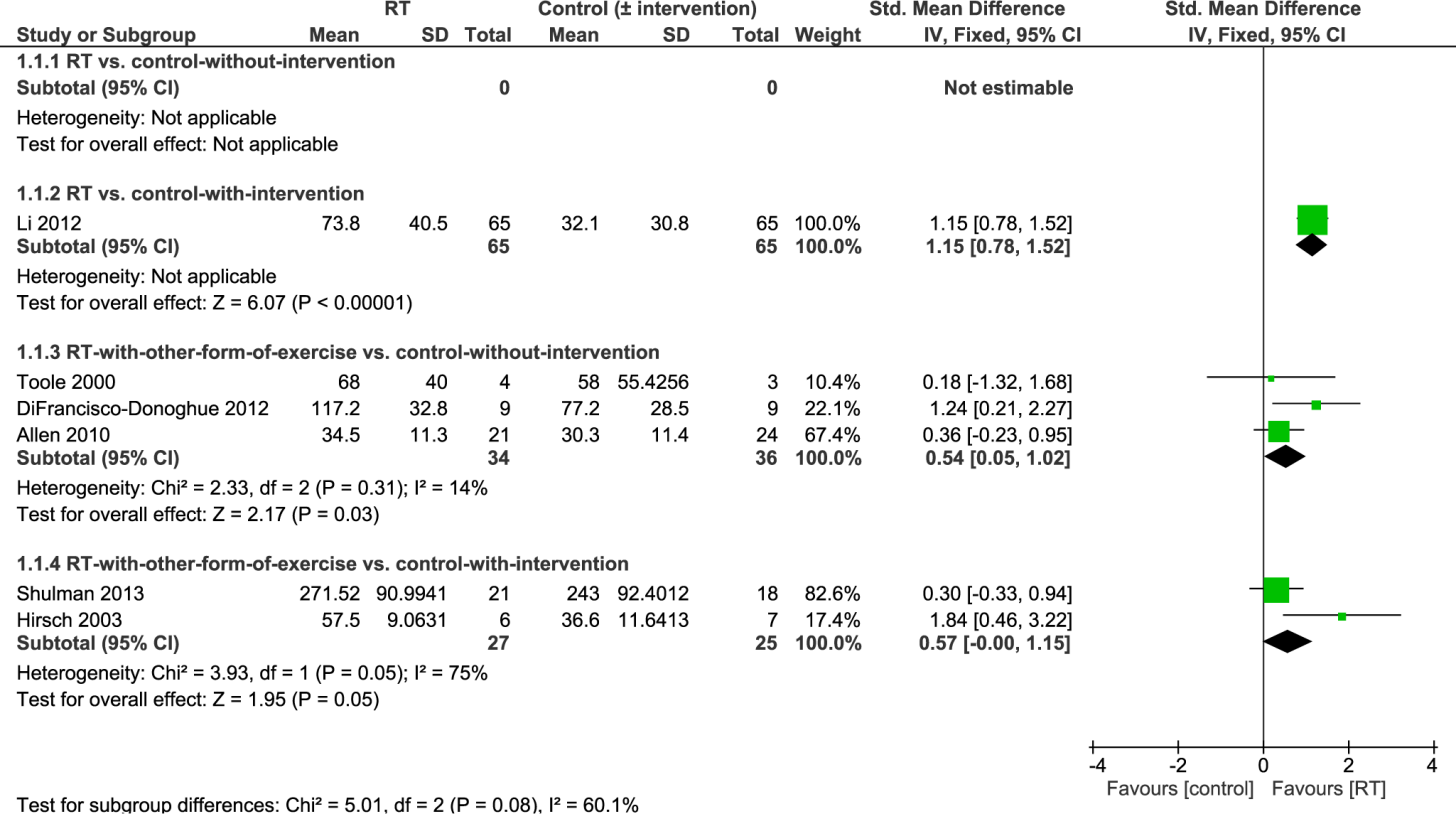
Subgroup analysis forest plot of comparison: RT vs. control-without-intervention, RT vs. control-with-intervention, RT with other form of exercise vs. control-without-intervention, RT with other form of exercise vs. control-with-intervention, using post-intervention values. Outcome: knee extension strength. CI = confidence interval; IV = inverse variance; SMD = standardized mean difference.

Results of the subgroup analysis according to differences in study design are summarized in Fig 5. The largest knee extension strength levels were found in people who performed RT for 24 weeks compared to people who underwent a stretching intervention (MD 41.70 Nm [95% CI 29.33, 54.07]) [40]. Knee extension strength was also significantly higher in people who undertook RT combined with another form of exercise (e.g. aerobic, balance training) compared to people who did not engage in any intervention after 6 weeks [38], 10 weeks [43] or 6 months [36] of training (SMD 0.54 [95% CI 0.05, 1.02]). There was significant heterogeneity (P = 0.05; I^2^ = 75%) in the fourth subgroup analysis (RT with other form of exercise vs. control-with-intervention). Using a random effects model knee extension strength was not significantly higher in people who undertook RT concurrently with balance or stretching exercise for 10 weeks [39] or 3 months [42] than in people who engaged in balance [39] or treadmill training [42] (SMD 0.95 [95% CI -0.54, 2.43]; data not displayed).

#### Muscle Strength: Knee Flexion

**Fig 6:**
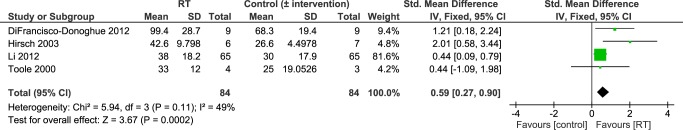
Primary analysis forest plot of comparison: RT vs. control-without/with-intervention, using post-intervention values, outcome: knee flexion strength. CI = confidence interval; IV = inverse variance; SMD = standardized mean difference.

Pooled data from four studies investigating the effects of RT on knee flexor strength [38–40,43] showed significantly higher knee flexion strength in people who had undergone an intervention that contained RT compared to controls-without-intervention or people who had undergone another intervention (SMD 0.59 [95%CI 0.27, 0.90], Fig 6). Although heterogeneity between studies was not statistically significant (P = 0.11) there may be a moderate level of heterogeneity (I^2^ = 49%).

**Fig 7:**
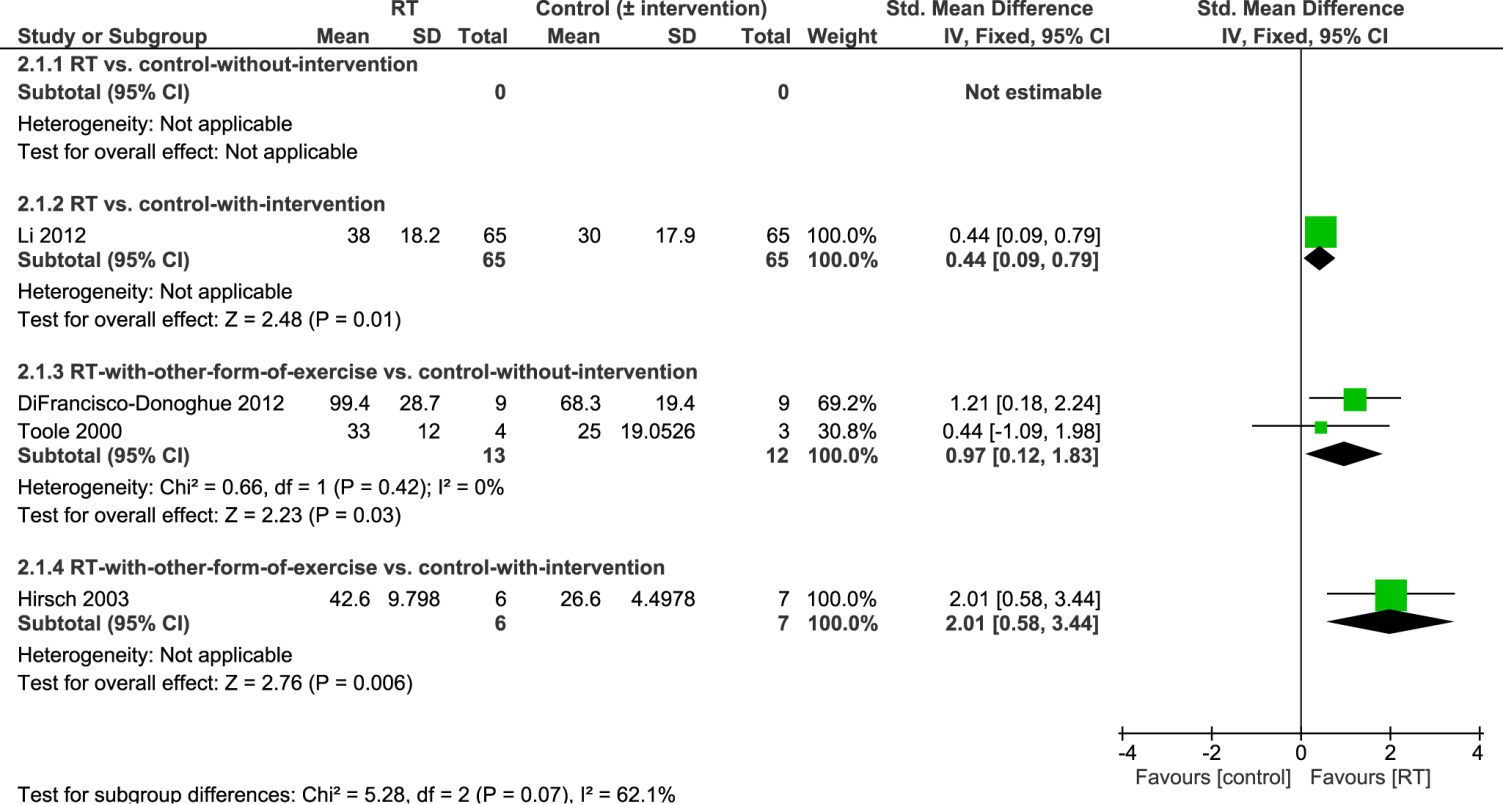
Subgroup analysis forest plot of comparison: RT vs. control-without-intervention, RT vs. control-with-intervention, RT with other form of exercise vs. control-without-intervention, RT with other form of exercise vs. control-with-intervention, using post-intervention values. Outcome: knee flexion strength. CI = confidence interval; IV = inverse variance; SMD = standardized mean difference.

Details of the subgroup analysis according to study design are summarized in Fig 7. Also the subgroup analysis revealed significantly higher knee flexion strength in people who had performed an intervention that contained RT. This was observed in people who performed RT for 24 weeks compared to people who engaged in a 24-week stretching intervention (MD 8 Nm [95% CI 1.79, 14.21]) [40], as well as in individuals who undertook simultaneous resistance and balance training over 10 weeks compared with individuals who performed balance training only (MD 16 kg [95% CI 7.48, 24.52]) [39], and in participants who underwent RT combined with aerobic training for six weeks [38] or RT with balance training for 10 weeks [43] compared to controls-without-intervention (SMD 0.97 [95% CI 0.12, 1.83]).

#### Muscle Strength: Leg Press

**Fig 8:**
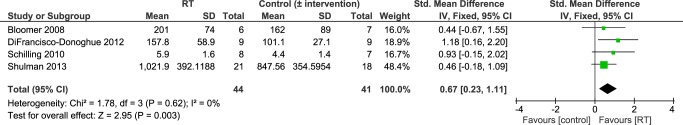
Primary analysis forest plot of comparison: RT vs. control-without/with-intervention, using post-intervention values, outcome: leg press strength. CI = confidence interval; IV = inverse variance; SMD = standardized mean difference.

Four studies [35,38,41,42] reported leg press strength as an outcome. Overall, pooled data revealed significant higher leg press strength in people who had undergone an intervention that contained RT compared to controls-without/with-intervention (SMD 0.67 [95%CI 0.23, 1.11]; Fig 8).

**Fig 9:**
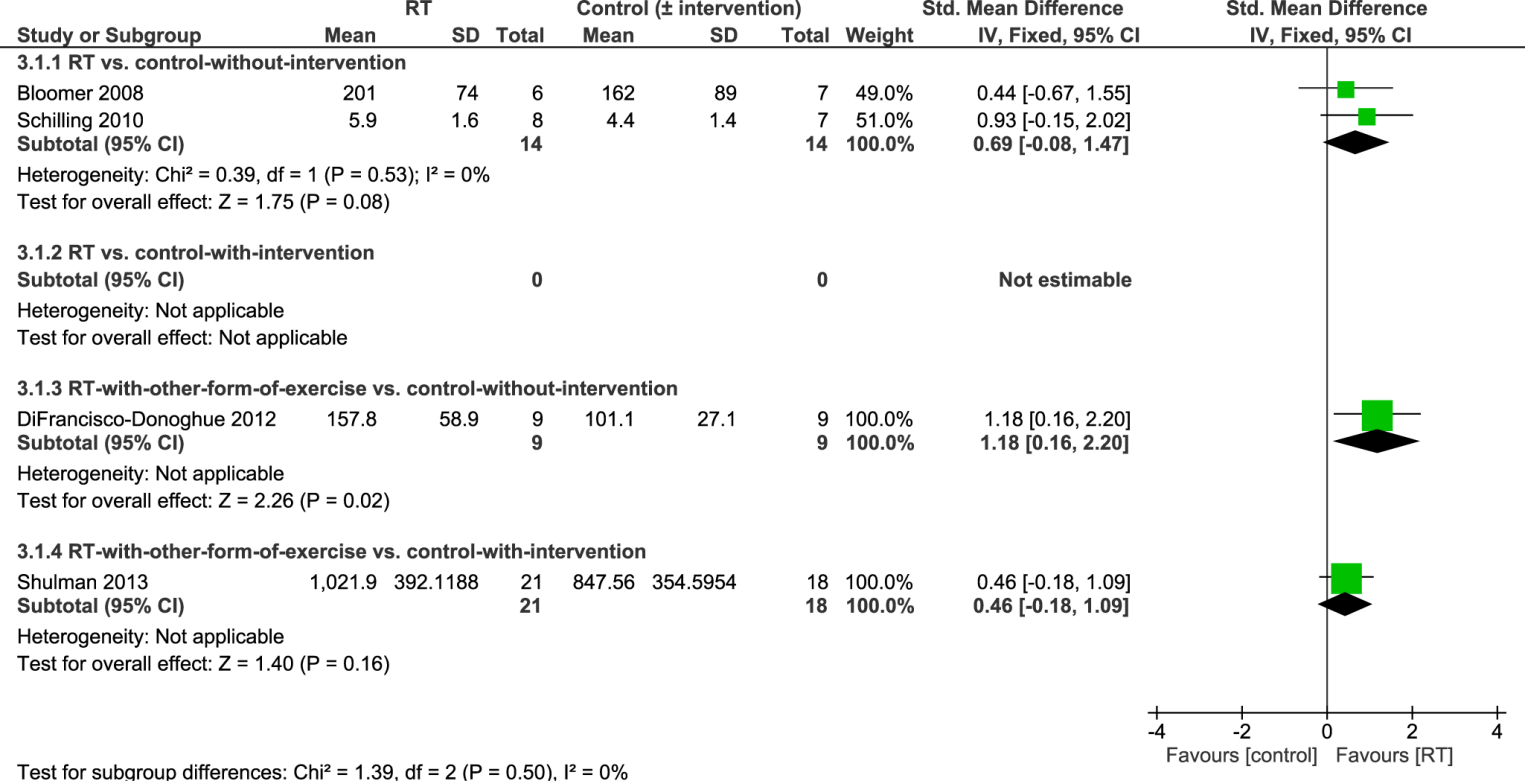
Subgroup analysis forest plot of comparison: RT vs. control-without-intervention, RT vs. control-with-intervention, RT with other form of exercise vs. control-without-intervention, RT with other form of exercise vs. control-with-intervention, using post-intervention values. Outcome: leg press strength. CI = confidence interval; IV = inverse variance; SMD = standardized mean difference.

Details of the subgroup analysis according to study design are presented in Fig 9. There was evidence from a single study [38] that leg press strength was significantly increased after 6 weeks of exercise that contained RT and aerobic exercise in people with PD compared to a control group without-intervention (MD 56.70 lb [95% CI 14.34, 99.06]). In contrast, Shulman et al. [42] found that 3-months RT and stretching did not lead to significantly larger leg press strength compared to treadmill training in people with PD (MD 174.34 lb [95% CI -60.10, 408.78]). Moreover, leg press strength was not significantly higher in participants who undertook RT in isolation for 8 weeks compared to a control group without-intervention [35,41] (SMD 0.69 [95% CI -0.08, 1.47]).

#### Muscle Strength: Other Outcome Measures

Hirsch et al. [39] found significant higher plantarflexion strength in PD patients who performed RT in combination with balance training over 10 weeks than in individuals who undertook balance training only (MD 23.6 kg [95% CI 13.00, 34.20]). With regards to ankle inversion strength [43] there was no significant difference found between participants who engaged in a 10-week RT-balance intervention and control participants without-intervention (MD 1 ft-lb [95% CI -6.07, 8.07]).

One study [37] reported significantly greater strength values in trunk flexion (MD 15.2 Nm [95% CI 11.79, 18.61]), trunk extension (MD 26.6 Nm [95% CI 22.72, 30.48]), and trunk rotation to the right (MD 8.91 Nm [95% CI 7.28, 10.54]) in people who performed resistance and aerobic training for 12 weeks compared to a control-without-intervention-group. Trunk rotation to the left did not show a significant difference between groups (MD -0.6 Nm [-2.48, 1.28]).

### Duration of Effects Post-Intervention

Three studies found that four weeks [37,39] to three months [40] after completion of the intervention, strength values were still significantly larger in the RT-group compared to controls-without-intervention or controls-with-intervention. At the end of a four-week follow-up period, people of the RT group showed higher knee extension strength (MD 16.8 kg [95% CI 4.46, 29.14]) [39], knee flexion strength (MD 11.8 kg [95% CI 1.79, 21.81] [39], plantarflexion strength (MD 15.9 kg [95% CI 3.06, 28.74] [39], trunk flexion strength (MD 4.3 Nm [95% CI 0.89, 7.71]) [37], trunk extension strength (MD 14.9 Nm [95% CI 11.02, 18.78]) [37], and rightwards-trunk-rotation strength (MD 8.37 Nm [95% CI 6.74, 10.00]; leftwards-trunk-rotation strength was not significant MD -2 Nm [95% CI -3.88, -0.12]) [37]. However, Bridgewater and Sharpe [37] noted that 23% of participants in the RT group continued exercising during the follow-up period while the remainder did not; hence, these results should be interpreted with caution. Li et al. [40] reported that the RT group maintained the level of strength during the three-month follow-up period (knee extension MD 15.8 Nm [95% CI 4.93, 26.67]; knee flexion MD 8.6 Nm [95% CI 2.96, 14.24]).

### Adverse Events

Only three studies [36,40,42] adequately reported exercise-induced complications, side-effects or adverse events. Li et al. [40] provided the greatest level of detail; they recorded adverse events over the course of the intervention (24 weeks) that occurred *during* exercise sessions and *outside* of exercise classes for each of the three intervention groups. *In class* 6.2% RT participants experienced a fall, 6.2% muscle soreness/pain, 4.6% dizziness and 4.6% symptoms of hypotension [40]; overall, the number of incidents per number of participants in the RT group was 0.22 versus 0.14 in the stretching group. *Outside of class*, 47.7% experienced a fall, 6.2% reported lower back pain, and <5% reported ankle sprain, symptoms of hypotension or chest pain [40]; the number of incidents per number of participants in the RT group was 0.63 versus 0.55 in the stretching group. Musculoskeletal damage or injuries following a fall (e.g. fracture) were not reported, neither was the context of a fall [40]. In the home-based study by Allen et al. [36] none of the participants experienced a fall *during* RT exercise and 14.3% reported back, shoulder or hip pain which appeared unrelated to the RT intervention. In Shulman et al. [42] no adverse events occurred *during* the RT sessions throughout the three-month intervention, however four people (18.2%) dropped out of the RT group due to medical reasons such as hypotension, joint pain and DBS. Although Toole et al. [43] did not report adverse events they stated that in the RT group 44% of trials during the balance pre-test (computerized dynamic posturography) led to a fall while no falls occurred in the post-test. Consequences of these falls and associated injuries were not described.

## Discussion

### Summary of findings

This systematic review and meta-analysis examines the overall effect of RT on different measures of muscular strength in people with PD. Overall, pooled data (between-group differences) indicated significantly higher muscular strength in people who had undergone an intervention that contained RT compared to controls-without-intervention (standard medical practice) or people who had undergone another intervention. Subgroup analysis according to study design revealed that RT combined with other forms of exercise (balance, aerobic) consistently led to significantly greater strength compared to controls-without-intervention but not compared to controls-with-intervention (balance, treadmill). RT alone did not result in significantly greater strength compared to controls-without-intervention although there was a positive trend. Due to the limited quality of the evidence, and the small sample size of most included studies, the current findings should be interpreted with caution.

### Participant Characteristics

The sample cohort included in this review is representative of an early stage PD population with low to moderate disease severity. Generally, it matches that prevalence of PD is higher in older age groups [3,4,46,47]. The majority of participants were male (62%) which reflects higher PD prevalence in men than in women with a male to female ratio of 1.46 [48]. However, a recent meta-analysis [4] identified higher prevalence in males than in females in the 50–59 age-group only. It is currently unknown if findings also apply to more advanced stages of the disease and it is unlikely that RT would be tolerable for patients in advanced stages considering movement and cognitive symptoms of PD.

It should be noted that few studies monitored and reported adverse events and no study described context and consequences of adverse events. Therefore, potential side effects are difficult to determine.

### Muscle Strength

Overall, the review suggests that exercise including RT is effective in improving muscle strength in people with PD. Considering that muscle weakness may be a primary symptom of PD [12], contributes to postural instability and gait difficulties [16,17], and has been identified as secondary cause for bradykinesia [18] this is an important insight and it emphasizes the role of RT in the treatment of PD.

However, this evidence arises from a large number of treatment comparisons and subgroup analysis, based on study design, revealed that there may be inconsistent intervention effects on different measures of strength (Figs 5, 7 and 9). It is important to highlight that only two small studies [35,41] have compared RT in isolation to a control group without intervention. All of the other studies have compared RT to other interventions and/or combined RT with another form of exercise (e.g. balance training, stretching, aerobic training). Studies that do not include a ‘non-exercise’ control group or that combine different interventions do not allow determination of which factors caused strength improvements. Notwithstanding, it may be unrealistic for PD patients to adopt a single form of exercise such as RT and many different types of exercise (treadmill training, dance, cueing, etc.) have shown beneficial effects on a variety of physical function measures [2]. Ultimately, it will be important to design an exercise treatment for people with PD that improves motor and non-motor complications across the disability spectrum and that allows patients to utilize the newly trained skills in their activities of daily living. There may be potential cumulative effects of different exercise treatments on a number of aspects of physical function and future research should focus on determining the most effective combination of interventions. RT should be included in such interventions because, as shown herein, RT is likely to improve muscular strength (see Figs 4, 6 and 8) especially in combination with another form of exercise (as suggested by pooled data of subgroup 3; Figs 5, 7 and 9). Moreover, it has been shown to improve leg muscle power [49], balance control [24,39] and disease severity [24,27,40]. It may also improve some aspects of gait (e.g. gait initiation) [40,50] although this has recently been questioned by two meta-analyses [24,25] which did not find significant gait improvements (gait speed, 6-minute-walking-test, timed-up-and-go-test) in the RT groups.

Interestingly, pooled data from two studies that compared RT in isolation to controls-without-intervention [35,41] (subgroup 1) did not show significantly greater strength in the RT groups (see Fig 9). While there was a positive (non-significant) trend towards greater strength in the RT groups compared to controls-without-intervention, these studies had a small sample size and a moderate to high risk-of-bias.

Differences in the chosen outcome measure, method of assessment, or the muscle group investigated may also play a role in the context of these results. Only one study found significantly greater *leg press* strength in the RT group (Fig 9), whereas pooled data of studies that assessed single-joint knee extension or flexion (Figs 5 and 7) showed significantly higher strength in the RT groups. A leg press strength assessment comprises a multi-joint movement which is more complex and involves more muscles than single-joint movements (e.g. knee extension/flexion). Hence, during a leg press test one does not only assess muscular strength of the quadriceps but also of the hip extensors. This corresponds to suggestions of previous studies that proximal muscles (i.e. hip extensors) show greater strength impairments than distal muscles (i.e. knee extensors/flexors) in people with PD [16,51]. Moreover, it has been observed that extensor muscles may be more affected by muscle weakness than flexor muscles in PD [13,52]. Taken together, if proximal and extensor muscles show greater strength deficits than distal and flexor muscles it might explain why leg press strength was not significantly higher in the RT groups (Fig 9) as opposed to knee flexor and knee extensor strength (Figs 5 and 7). However, results of this review refer to post-intervention data; muscles have already been trained and one would therefore assume that imbalanced strength deficits across proximal-distal or extensor-flexor muscles may have been evened out. This raises questions whether muscles that are more affected by weakness (extensors and proximal muscles) are as trainable compared to others. It may be necessary to focus a RT program for people with PD on muscle groups that are more prone to weakness in order to balance out the uneven distribution of muscle strength.

Overall though, these reflections are speculative as data available to date are too sparse to draw a definitive conclusion. Nonetheless, results herein show for the first time that strength increases following RT in people with PD may not be as consistent as suggested previously [15,22,23,28], but that they might vary with muscle group or training mode.

In addition, evidence regarding durability of strength improvements in response to RT in people with PD is inconclusive. Available data do not allow assessing whether effects might habituate over time. All studies ran over a short- to medium-term time period of six weeks to six months (see Table 1) and only three studies provided follow-up data [37,39,40] which were not possible to pool. Data from these individual studies [37,39,40] suggest that it is possible to maintain improved strength levels for up to three months after completion of the intervention but potential changes afterwards are unknown to date. Also, it is not clear whether strength increases stagnate over the course of a medium- to long-term intervention. However, since it is clinically of interest to incorporate RT long-term in the treatment of a chronic and progressive condition such as PD, there is a strong need for long-term studies that investigate durability of beneficial effects such as strength and mobility improvements. Corcos and colleagues [27], for example, showed that strength might not increase consistently over the course of a two–year progressive RT intervention in people with PD. Rather, strength increased within the first six months of the intervention and then plateaued for the remaining 18 months. However, these findings need to be confirmed in future RCTs that include a ‘non-exercise’ control arm [53].

Methodologically, it is important to highlight the heterogeneity in strength measurements utilized in the included studies (testing protocol, muscle groups, reported units) which makes comparability of trials and interpretation of findings difficult. There is certainly need for standardization of strength assessments in future studies in order to improve comparability of studies. We recommend that, where feasible, future RT studies utilize isokinetic dynamometry for strength assessment and that specifications are kept consistent across studies (velocity, seating position, muscle group, unit in Nm). Otherwise, 1RM testing has been shown as an appropriate assessment of strength in people with PD [54] and it might be easier to conduct in a clinical setting. Moreover, it is worthwhile mentioning that all included studies only analyzed maximal voluntary contraction. Future studies should also analyze the effects of RT on other strength related measures such as rate of force development. This would provide valuable information in order to improve future interventions and maximize beneficial effects on other outcomes related to physical function.

### Training Dose

In this review, high variation was evident across studies in the training durations, frequencies, modes, volumes, intensities and progression. This makes it difficult to identify characteristics of effective RT interventions and to provide evidence-based guidelines at the present time. It clearly demonstrates the difficulty in finding a best-practice RT program for people with PD and highlights the need for more research into training dose. As discussed in the paragraphs above, this meta-analysis suggests that a combination of RT with other forms of exercise may be most effective to increase strength in people with PD.

Some guidelines for RT have been provided previously [28] which this meta-analysis generally supports. We also recommend utilizing RT interventions for healthy elderly as a guide for prescribing RT to people with PD. In the elderly, for example, it has been shown that high intensity RT may be more effective in improving strength than low intensity programs whereas training frequency and volume may not be such a crucial factor in influencing the magnitude of strength improvements [55]. However, it has also been shown that the participants’ health status and physical function impacts effect size [55] which is important to consider for a PD study population. Moreover, previous findings in a PD population indicate that eccentric RT resulted in greater strength increases than non-eccentric RT in people with PD [56,57]; these findings should be considered for the development of future RCTs.

Finally, it is important to note that *reporting* of acute training variables was heterogeneous across studies as well. Duration, frequency and mode were reasonably well documented in all studies, although more details could be provided for training mode (e.g. seat/body/joint position on machines). However, volume, intensity and progression were reported in distinctly different ways. Often it was not clear in what way the number of repetitions were increased during the intervention or at what intensity levels participants trained and how the program was progressed for each individual. Moreover, some studies also provided details on more variables that are relevant for the overall training dose. These included duration of each training session [36,39,40,43], rest intervals [38,39] and movement velocity [39,41,43]. We suggest that future studies report clearly on each training variable in order to improve comparability between studies.

### Comparison to Other Reviews

A number of narrative [15,28] and systematic [22–25] reviews have previously examined the benefits of RT for people with PD. While these previous reviews focused their analyzes on the effects of RT on different health related measures of physical function (e.g. strength, mobility, balance, gait) the current review and meta-analysis investigated the effects of RT on measures of muscular strength in detail and provides a differentiated analysis with respect to various study designs and outcome measures. Generally in agreement with the other RT reviews, our meta-analysis (primary analysis results) also suggests that exercise incorporating RT is effective in improving muscular strength in people with PD. However, subgroup analysis based on study design demonstrated that strength increases following RT may not occur in all muscle groups equally and that not all RT interventions may lead to significant strength improvements in people with PD. This review also emphasizes the lack of studies that compared RT alone with a ‘non-exercise’ control group.

### Quality of Evidence

We found a relatively high risk of bias across all reviewed studies which indicates a limited quality of evidence. Data used in this meta-analysis are mainly from studies with a moderate-high risk of bias (see Figs 2 and 3). However, pooled effect estimates were consistent for all outcome measures with similar magnitudes (see Figs 4, 6 and 8) and generally in agreement with previous reviews [22–25]. Also results of the subgroup analyses showed a positive trend in favor of RT, although they were not statistically significant (e.g. subgroup 4: RT with other form of exercise vs. control-with-intervention). It is important to note that the non-significant results of our subgroup analysis must not be taken as evidence for no-effect or no-difference between groups. On the contrary, because of the limited evidence, in terms of the quality and the quantity of the included studies, conclusions are not definitive; thus, appropriately powered RCTs that include a non-exercise control arm are required. Our analysis also highlights many areas of methodological uncertainty of RT studies and, therefore, guides the design of future trials.

### Limitations and Future Studies

We undertook an exhaustive search based on multiple electronic databases and supplementary sources. Nonetheless, we acknowledge that other relevant studies in the grey literature or in other languages may have been overlooked. Bias from selective reporting of results and from allocation concealment was difficult to determine as the published reports did not provide sufficient details for judgment. Since much was unknown about the quality of most included studies, it impacts on conclusions drawn from this review which are not definitive. We acknowledge that we only investigated effects of RT on strength but not on other outcome measures related to physical function, mobility or non-motor symptoms that may be of interest for treatment of PD (for review see [22–25]). However, we were able to conduct an additional subgroup analysis and this approach suggested that there may be inconsistent effects of RT on measures of muscular strength in people with PD which, in turn, highlights the need for future research.

We recommend that future studies comprise an appropriately powered RCT with adequate sequence generation and allocation concealment, and employ methods to limit detection, attrition and reporting bias. Second, RT interventions should be carefully designed with regards to acute training variables based on a sound physiological rationale (for review see [15,28]) and should aim to investigate a best-practice RT for the treatment of PD over short- and long-term. Third, active monitoring of pre-defined adverse events should be undertaken in future RT studies and reported accordingly. Fourth, measurement of strength should be standardized across studies and strength related measures other than maximal voluntary contraction (e.g. rate of force development) should also be recorded. Fifth, future trials should include participants of all stages of the disease (RT programs will have to be amended accordingly to make it feasible for patients in more advanced stages of the disease) with respect to generalizability of findings towards the overall PD population. Finally, assessment of disease severity should be standardized across studies using the MDS-UPDRS [58] as a subjective, assessor-rated scale; there is a strong need for additional objective measurements of disease severity.

### Conclusion

Overall, the current evidence suggests that exercise interventions that contain RT are effective in improving muscular strength in people with PD compared with no exercise. However, depending on muscle group and/or training dose RT may not be superior to other types of exercise (e.g. aerobic). Results indicate that an intervention that combines RT with another form of exercise may be most effective. There are not enough data available yet to confirm evidence-based guidelines for prescribing RT to PD patients.

These conclusions are based on limited methodological quality and relatively small sample sizes in the reviewed studies, and are not definitive. Well reported RCTs in this area are required in order to develop a best-practice RT intervention for people with PD. Until better evidence is available, health professionals are advised to incorporate RT of moderate to high intensity in an exercise treatment that combines different exercise modalities (e.g. aerobic exercise and RT) and that is designed progressively over a mid- to long-term time period.

## Supporting Information

S1 Appendix PRISMA checklist. (PDF)

S2 Appendix Search Strategy. (PDF)

S3 Appendix Excluded Studies. (PDF)
