# Supplementary material for: Effects of Resistance Training on Measures of Muscular Strength in People with Parkinson’s Disease: A Systematic Review and Meta-Analysis
Source: PLoS One. 2015 Jul 6;10(7):e0132135. doi: 10.1371/journal.pone.0132135 (PMC4492705; doi:10.1371/journal.pone.0132135)
Supplement: S2 Appendix — (PDF) [file pone.0132135.s002.pdf]

Search Strategy

Database: Medline (via Ovid SP)

	Search term
1	exp Parkinson's disease/
2	Parkinson$.ti. or Parkinson$.ab.
3	#1 or #2
4	(resistance training or resistance exercise).mp. or resistance training/
5	(strength training or strength* exercise).mp. or strength training/
6	exercise.mp. or Exercise/
7	exercise therapy.mp. or Exercise Therapy/
8	exercise tolerance.mp. or exercise tolerance/ or exercise test/
9	(physiotherapy or physical therapy).mp.
10	rehabilitation.mp. or rehabilitation/
11	sports.mp. or sports/
12	weight lifting.mp. or weight lifting/
13	isometric contraction/ or isotonic contraction.mp.
14	((training or conditioning) adj3 (intervention$ or protocol$ or program$ or activit$ or regim$)).mp.
15	(exercise adj3 (train$ or intervention$ or protocol$ or program$ or therap$ or activit$ or regim$)).mp.
16	(muscle strengthening or progressive resist$).mp.
17	((weight or strength$ or resist$) adj (train$ or lift$ or exercise$)).mp.
18	((isometric or isotonic or eccentric or concentric) adj (contraction$ or exercise$)).mp.
19	or/4-18
20	randomized controlled trial.pt.
21	randomized controlled trials.mp. or Randomized Controlled Trial/
22	controlled clinical trial.pt.
23	controlled clinical trials.mp. or controlled clinical trial/
24	random allocation.mp. or Random Allocation/
25	single-blind method.mp. or Single-Blind Method/
26	double-blind method.mp. or double-blind Method/

27 clinical trial.pt.

28 exp clinical trial/

29 (clin$ adj5 trial$).mp.

30 (single adj5 (blind$ or mask$)).mp.

31 (double adj5 (blind$ or mask$)).mp.

32 placebos.mp. or Placebos/

33 placebo$.mp. or Placebos/

34 random$.mp.

35 research design.mp. or Research Design/

36 multicenter study.pt.

37 intervention studies.mp. or Intervention Studies/

38 cross-over studies.mp. or Cross-Over Studies/

39 control$.tw.

40 alternat$ treatment.tw.

41 Comparative Study/

42 exp evaluation studies/

43 follow-up studies.mp. or Follow-Up Studies/

44 prospective studies.mp. or Prospective Studies/

45 prospective.tw.

46 counterbalance$.tw.

47 versus.tw.

48 or/20-47

49 #3 and #19 and #48

Database: Cochrane Library (via Wiley Online)

	**Search term**
1	[mh "Parkinson Disease"]
2	Parkinson*:ti or Parkinson*:ab
3	#1 or #2
4	resistance training:kw or resistance exercise:kw or [mh "resistance training"]
5	strength training:kw or strength* exercise:kw or [mh "strength training"]
6	exercise:kw or [mh Exercise]
7	exercise therapy:kw or [mh "Exercise Therapy"]
8	exercise tolerance:kw or [mh “exercise tolerance"] or [mh "exercise test"]
9	(physiotherapy or physical therapy):kw
10	rehabilitation:kw or [mh rehabilitation]
11	sports:kw or [mh sports]
12	weight lifting:kw or [mh "weight lifting"]
13	[mh "isometric contraction"] or isotonic contraction:kw
14	((training or conditioning) near/3 (intervention* or protocol* or program* or activit* or regim*)):kw
15	(exercise near/3 (train* or intervention* or protocol* or program* or therap* or activit* or regim*)):kw
16	(muscle strengthening or progressive resist*):kw
17	((weight or strength* or resist*) near (train* or lift* or exercise*)):kw
18	((isometric or isotonic or eccentric or concentric) near (contraction* or exercise*)):kw
19	{or #4-#18}
20	“randomized controlled trial”:pt
21	“randomized controlled trials”:kw or [mh "Randomized Controlled Trial"]
22	“controlled clinical trial”:pt
23	controlled clinical trials:kw or [mh "controlled clinical trial"]
24	random allocation:kw or [mh "Random Allocation"]
25	single-blind method:kw or [mh "Single-Blind Method"]
26	double-blind method:kw or [mh "double-blind Method"]
27	clinical trial:pt
28	[mh "clinical trial"]

29 (clin* near/5 trial*):kw

30 (single near/5 (blind* or mask*)):kw

31 (double near/5 (blind* or mask*)):kw

32 placebos:kw or [mh Placebos]

33 placebo*:kw or [mh Placebos]

34 random*:kw

35 research design:kw or [mh "Research Design"]

36 multicenter study:pt

37 intervention studies:kw or [mh "Intervention Studies"]

38 cross-over studies:kw or [mh "Cross-Over Studies"]

39 control*

40 alternat* treatment

41 [mh "Comparative Study"]

42 [mh "evaluation studies"]

43 follow-up studies:kw or [mh "Follow-Up Studies"]

44 prospective studies:kw or [mh "Prospective Studies"]

45 prospective

46 counterbalance*

47 versus

48 {OR #20-#47}

49 #3 and #19 and #48

Database: CINAHL (via Ebscohost)

	Search term
1	(MH "Parkinson Disease")
2	TI Parkinson* OR AB Parkinson*
3	S1 OR S2
4	TX resistance training OR TX resistance exercise OR (MH "Muscle Strengthening+")
5	TX strength* training OR TX strength* exercise OR (MH "Muscle Strengthening+")
6	(MH "Exercise+") OR TX exercise
7	(MH "Therapeutic Exercise+") OR TX exercise therapy
8	(MH "Exercise Tolerance+") OR (MH "Exercise Test+") OR TX exercise tolerance
9	(MH "Physical Therapy+") OR TX physical therapy OR TX physiotherapy
10	(MH "Rehabilitation+") OR TX rehabilitation
11	(MH "Sports+") OR TX sports
12	(MH "Weight Lifting") OR TX weight lifting
13	(MH "Isometric Contraction") OR (MH "Isotonic Contraction+") OR TX isotonic contraction OR TX isometric contraction
14	TX ((training or conditioning) N3 (intervention* or protocol* or program* or activit* or regim*))
15	TX (exercise N3 (train* or intervention* or protocol* or program* or therap* or activit* or regim*))
16	TX muscle strengthening OR TX progressive resist*
17	TX (weight or strength* or resist*) N5 (train* or lift* or exercise*)
18	TX (isometric or isotonic or eccentric or concentric) N5 (contraction* or exercise*)
19	S4 OR S5 OR S6 OR S7 OR S8 OR S9 OR S10 OR S11 OR S12 OR S13 OR S14 OR S15 OR S16 OR S17 OR S18
20	PT randomized controlled trial
21	(MH "Randomized Controlled Trials") OR TX randomized controlled trials
22	PT controlled clinical trial
23	(MH "Clinical Trials+") OR TX controlled clinical trial
24	(MH "Random Assignment") OR TX random allocation
25	(MH "Single-Blind Studies") OR TX single-blind method
26	(MH "Double-Blind Studies") OR TX double-blind method

27 PT clinical trial

28 TX clin* N5 trial*

29 TX single N5 (blind* or mask*)

30 TX double N5 (blind* or mask*)

31 (MH "Placebos") OR TX placebos

32 TX placebo* OR (MH "Placebos")

33 TX random*

34 (MH "Study Design+") OR TX research design

35 PT multicenter study

36 (MH "Experimental Studies+") OR TX intervention studies

37 (MH "Crossover Design") OR TX cross-over studies

38 TX control*

39 TX alternat* treatment

40 (MH "Comparative Studies")

41 (MH "Evaluation Research+")

42 (MH "Prospective Studies+") OR TX follow-up studies OR TX prospective studies

43 TX prospective

44 TX counterbalance*

45 TX versus

46 S20 OR S21 OR S22 OR S23 OR S24 OR S25 OR S26 OR S27 OR S28 OR S29 OR S30 OR S31 OR S32 OR S33 OR S34 OR S35 OR S36 OR S37 OR S38 OR S39 OR S40 OR S41 OR S42 OR S43 OR S44 OR S45

47 S3 AND S19 AND S46

Database: Embase (via Elsevier)

	Search term
1	'parkinson disease'/exp AND [embase]/lim
2	parkinson*:ab,ti AND [embase]/lim
3	#3.1 OR #3.2
4	'resistance training'/de OR 'resistance exercise'/de AND [embase]/lim
5	'strength training'/de OR 'strength exercise' AND [embase]/lim
6	'exercise'/de AND [embase]/lim
7	'exercise therapy'/de AND [embase]/lim
8	'exercise tolerance'/de OR 'exercise test'/de AND [embase]/lim
9	'physiotherapy'/de OR 'physical therapy'/de AND [embase]/lim
10	'rehabilitation'/de AND [embase]/lim
11	'sports'/de AND [embase]/lim
12	'weight lifting'/de AND [embase]/lim
13	'isometric contraction'/de OR 'isotonic contraction'/de AND [embase]/lim
14	(training OR conditioning) NEAR/3 (intervention* OR protocol* OR program* OR activit* OR regim*) AND [embase]/lim
15	exercise NEAR/3 (train* OR intervention* OR protocol* OR program* OR therap* OR activit* OR regim*) AND [embase]/lim
16	'muscle strengthening'/de OR 'muscle strengthening' OR progressive NEAR/3 resist* AND [embase]/lim
17	(weight* OR strength* OR resist*) NEAR/3 (train* OR lift* OR exercise*) AND [embase]/lim
18	(isometric OR isotonic OR eccentric OR concentric) NEAR/3 (contraction* OR exercise*) AND [embase]/lim
19	#19.1 OR #19.2 OR #19.3 OR #19.4 OR #19.5 OR #19.6 OR #19.7 OR #19.8 OR #19.9 OR #19.10 OR #19.11 OR #19.12 OR #19.13 OR #19.14 OR #19.15
20	'randomized controlled trial':it AND [embase]/lim
21	'randomized controlled trials'/de OR 'randomized controlled trial'/de AND [embase]/lim
22	'controlled clinical trial':it AND [embase]/lim
23	'controlled clinical trials'/de OR 'controlled clinical trial'/de AND [embase]/lim
24	'random allocation'/de AND [embase]/lim
25	'single-blind method'/de AND [embase]/lim

26 'double-blind method'/de AND [embase]/lim

27 'clinical trial':it AND [embase]/lim

28 'clinical trial'/exp AND [embase]/lim

29 clin* NEAR/5 trial* AND [embase]/lim

30 single NEAR/5 (blind* OR mask*) AND [embase]/lim

31 double NEAR/5 (blind* OR mask*) AND [embase]/lim

32 'placebos'/de AND [embase]/lim

33 placebo* AND [embase]/lim

34 random* AND [embase]/lim

35 'research design'/de AND [embase]/lim

36 'multicenter study':it AND [embase]/lim

37 'intervention studies'/de AND [embase]/lim

38 'cross-over studies'/de AND [embase]/lim

39 control* AND [embase]/lim

40 alternat* NEAR/3 treatment AND [embase]/lim

41 'comparative study'/de AND [embase]/lim

42 'evaluation studies'/de AND [embase]/lim

43 'follow-up studies'/de AND [embase]/lim

44 'prospective studies'/de AND [embase]/lim

45 prospective AND [embase]/lim

46 counterbalance* AND [embase]/lim

47 versus AND [embase]/lim

48 #48.1 OR #48.2 OR #48.3 OR #48.4 OR #48.5 OR #48.6 OR #48.7 OR #48.8 OR #48.9 OR #48.10 OR #48.11 OR #48.12 OR #48.13 OR #48.14 OR #48.15 OR #48.16 OR #48.17 OR #48.18 OR #48.19 OR #48.20 OR #48.21 OR #48.22 OR #48.23 OR #48.24 OR #48.25 OR #48.26 OR #48.27 OR #48.28

49 #49.3 AND #49.19 AND #49.48

Database: SPORTDiscus (via Ebscohost)

	**Search term**
1	TX parkinson's disease
2	TI parkinson* OR AB parkinson*
3	S1 OR S2
4	TX resistance training OR TX resistance exercise
5	TX strength training OR TX strength* exercise
6	TX exercise OR SU exercise OR KW exercise
7	TX exercise therapy OR SU exercise therapy OR KW exercise therapy
8	TX exercise tolerance OR TX exercise test
9	TX physiotherapy OR TX physical therapy
10	TX rehabilitation
11	TX sports
12	TX weight lifting
13	TX isometric contraction OR TX isotonic contraction
14	TX ((training or conditioning) N3 (intervention* or protocol* or program* or activit* or regim*))
15	TX (exercise N3 (train* or intervention* or protocol* or program* or therap* or activit* or regim*))
16	TX muscle strengthening OR TX progressive resist*
17	TX (weight or strength* or resist*) N5 (train* or lift* or exercise*)
18	TX (isometric or isotonic or eccentric or concentric) N5 (contraction* or exercise*)
19	S4 OR S5 OR S6 OR S7 OR S8 OR S9 OR S10 OR S11 OR S12 OR S13 OR S14 OR S15 OR S16 OR S17 OR S18
20	TX randomized controlled trial OR TX randomized controlled trials
21	TX controlled clinical trial OR TX controlled clinical trials
22	TX random allocation OR TX random assignment
23	TX single-blind method OR TX single-blind study
24	TX double-blind method OR TX double-blind study
25	TX clinical trial OR TX clinical trials
26	TX clin* N5 trial*
27	TX single N5 (blind* or mask*)

28 TX double N5 (blind* or mask*)

29 TX placebos OR TX placebo*

30 TX random*

31 TX research design OR TX study design

32 TX multicenter study

33 TX interventions OR TX intervention study OR TX intervention studies OR TX intervention

34 TX cross-over studies OR TX crossover trials OR TX crossover design OR TX crossover studies OR cross-over trials OR cross-over design

35 TX control*

36 TX alternat* treatment

37 TX comparative studies OR TX comparative study

38 TX evaluation methodology OR TX evaluation studies OR TX evaluation research

39 TX follow-up studies OR TX follow-up study

40 TX prospective studies OR TX prospective study

41 TX prospective

42 TX counterbalance*

43 TX versus

44 S20 OR S21 OR S22 OR S23 OR S24 OR S25 OR S26 OR S27 OR S28 OR S29 OR S30 OR S31 OR S32 OR S33 OR S34 OR S35 OR S36 OR S37 OR S38 OR S39 OR S40 OR S41 OR S42 OR S43

45 S3 AND S19 AND S44
