# Supplementary material for: Effects of Resistance Training on Measures of Muscular Strength in People with Parkinson’s Disease: A Systematic Review and Meta-Analysis
Source: PLoS One. 2015 Jul 6;10(7):e0132135. doi: 10.1371/journal.pone.0132135 (PMC4492705; doi:10.1371/journal.pone.0132135)
Supplement: S3 Appendix — (PDF) [file pone.0132135.s003.pdf]

Excluded studies after full text assessment

Study	Reason for exclusion
A'Campo, L.E., N.G. Spliethoff-Kamminga, and R.A. Roos, *An evaluation of the patient education programme for Parkinson's disease in clinical practice.* International Journal of Clinical Practice, 2011. **65**(11): p. 1173-1179.	• The education programme does not include resistance training<br>• No measure of muscular strength
Allen, N.E., J. Song, C. Sherrington, S.R. Lord, V.S.C. Fung, J.C.T. Close, S.S. Paul, S.D. O'Rourke, S.M. Murray, and C.G. Canning, *Predictors of adherence to an exercise program in people with Parkinson's disease.* Movement Disorders, 2013. **28**: p. S157.	• No measure of muscular strength<br>• No full text available
Almangour W, Calvalido A, Pauwels C, Hutin E, Bayle N, et al. (2014) Effects of an intensive physical therapy program on a marker of Parkinson's disease: Alternate forearm pronation/supination movements of large vs small amplitude. Annals of Physical and Rehabilitation Medicine 57: e362-e363.	No full text available
Alvarez, M.V., P.M. Grogan, and M. Rodriguez, *Large amplitude exercise ('ThinkBIG') improves motor dysfunction in Parkinson's disease patients.* Movement Disorders, 2010. **25**: p. S254.	No full text available
Ashburn, A., L. Fazakarley, C. Ballinger, R. Pickering, L.D. McLellan, and C. Fitton, *A randomised controlled trial of a home based exercise programme to reduce the risk of falling among people with Parkinson's disease.* Journal of Neurology, Neurosurgery & Psychiatry, 2007. **78**(7): p. 678-84.	No measure of muscular strength
Ayan, C. and J. Cancela, *Feasibility of 2 different water-based exercise training programs in patients with Parkinson's disease: a pilot study.* Archives of Physical Medicine & Rehabilitation, 2012. **93**(10): p. 1709-14.	No measure of muscular strength
Ayán, C. and J.M. Cancela, *Effects of aquatic exercise on persons with Parkinson's disease: A preliminary study. / Effets de l'exercice aquatique sur les malades de Parkinson, étude préliminaire.* Science & Sports, 2012. **27**(5): p. 300-304.	No measure of muscular strength
Ayan, C., J.M. Cancela, A. Gutierrez-Santiago, and I. Prieto, *Effects of two different exercise programs on gait parameters in individuals with Parkinson's disease: A pilot study.* Gait and Posture, 2013.	No measure of muscular strength
Baatile, J., W.E. Langbein, F. Weaver, C. Maloney, and M.B. Jost, *Effect of exercise on perceived quality of life of individuals with Parkinson's disease.* Journal of Rehabilitation Research & Development, 2000. **37**(5): p. 529-34.	No resistance training

Study	Reason for exclusion
Bartolo, M., M. Serrao, C. Tassorelli, R. Don, A. Ranavolo, F. Draicchio, C. Pacchetti, S. Buscone, A. Perrotta, A. Furnari, P. Bramanti, L. Padua, F. Pierelli, and G. Sandrini, *Four-week trunk-specific rehabilitation treatment improves lateral trunk flexion in Parkinson's disease.* Movement Disorders, 2010. **25**(3): p. 325-31.	No measure of muscular strength
Batistela, R.A., A.K.G. Prado, E. Lirani-Silva, F.A. Barbieri, R. Vitorio, P.C.R. Santos, L.C. Morais, and L.T.B. Gobbi, *Effects of multimodal exercise program on functional capacity in people with Parkinson's disease.* Movement Disorders, 2011. **26**: p. S120.	No full text available
Blackinton, M.T., L. Summerall, and K. Waguespack, *Tertiary prevention in Parkinson disease: results from a preliminary study.* Neurology Report, 2002. **26**(3): p. 160-165.	No measure of muscular strength,
Boehm, R.L., Q.J. Almeida, and P. Knobl, *Sensory attention focused exercise in Parkinson's disease: A randomized double-crossover trial.* Movement Disorders, 2011. **26**: p. S331-S332.	No full text available
Bridgewater, K.J. and M.H. Sharpe, *Trunk muscle performance in early Parkinson's disease.* Physical Therapy, 1998. **78**(6): p. 566-576.	Not an intervention study
Caglar, A.T., H.N. Gurses, F.K. Mutluay, and G. Kiziltan, *Effects of home exercises on motor performance in patients with Parkinson's disease.* Clinical Rehabilitation, 2005. **19**(8): p. 870-7.	• No measure of muscular strength<br>• No resistance training
Cakit, B.D., M. Saracoglu, H. Genc, H.R. Erdem, and L. Inan, *The effects of incremental speed-dependent treadmill training on postural instability and fear of falling in Parkinson's disease.* Clinical Rehabilitation, 2007. **21**(8): p. 698-705.	• No measure of muscular strength<br>• No resistance training
Canning, C.G., N.E. Allen, C.M. Dean, L. Goh, and V.S. Fung, *Home-based treadmill training for individuals with Parkinson's disease: a randomized controlled pilot trial.* Clinical Rehabilitation, 2012. **26**(9): p. 817-26.	• No measure of muscular strength<br>• No resistance training
Canning, C.G., N.E. Allen, C.M. Dean, L. Goh, and V.S.C. Fung, *Minimally-supervised treadmill training for individuals with Parkinson's disease: A randomized controlled trial.* Neurorehabilitation and Neural Repair, 2012. **26**(6): p. 703-704.	No full text available
Canning, C.G., C. Sherrington, S.R. Lord, J.C.T. Close, G. Heller, S. Heritier, K. Howard, N.E. Allen, S.S. Paul, S.M. Murray, S.D. O'Rourke, and V.S.C. Fung, *Exercise for falls prevention in Parkinson's disease: A randomised controlled trial.* Movement Disorders, 2013. **28**: p. S158.	No full text available
Capato, T. and M.E. Piemonte, *Global motor training with rythmical auditory cues improve and maintain balance control in Parkinson's disease (PD) patients.* Movement Disorders, 2010. **25**: p. S292.	No full text available

Study	Reason for exclusion
Carne, W., D.X. Cifu, P. Marcinko, M. Baron, T. Pickett, A. Qutubuddin, V. Calabrese, P. Roberge, K. Holloway, and B. Mutchler, *Efficacy of multidisciplinary treatment program on long-term outcomes of individuals with Parkinson's disease.* Journal of Rehabilitation Research & Development, 2005. **42**(6): p. 779-86.	• Not an intervention study<br>• No measure of muscular strength
Cheon, S.M., H.R. Sung, B.K. Chae, and J.W. Kim, *Effects of different exercises in the patients with Parkinson's disease.* Parkinsonism and Related Disorders, 2012. **18**: p. S154.	No full text available
Cheon, S.M., H.R. Sung, B.K. Chae, H.J. Ryu, and J.W. Kim, *Benefits of exercise in Parkinson's disease.* Movement Disorders, 2012. **27**: p. S111.	No full text available
Chien HF, Chen J, Souza CO, Voos MC, Frqancato DV, et al. (2014) The effect of muscular strenghtening exercise in motor function and balance in Parkinson's disease patients. Movement Disorders 29: S230-S231.	No full text available
Christofoletti, G., F. Beinotti, G. Borges, and B.P. Damasceno, *Physical therapy improves the balance of patients with Parkinson's disease: A randomized controlled trial.* Parkinsonism and Related Disorders, 2010. **16**: p. S58.	No full text available
Combs, S.A., M.D. Diehl, C. Chrzastowski, N. Didrick, B. McCoin, N. Mox, W.H. Staples, and J. Wayman, *Community-based group exercise for persons with Parkinson disease: a randomized controlled trial.* Neurorehabilitation, 2013. **32**(1): p. 117-24.	No measure of muscular strength
Combs, S.A., M.D. Diehl, W.H. Staples, L. Conn, K. Davis, N. Lewis, and K. Schaneman, *Boxing training for patients with parkinson disease: a case series.* Physical Therapy, 2011. **91**(1): p. 132-142.	No measure of muscular strength
Comella, C.L., G.T. Stebbins, N. Brown-Toms, and C.G. Goetz, *Physical therapy and Parkinson's disease: a controlled clinical trial.* Neurology, 1994. **44**(3 Pt 1): p. 376-8.	• No resistance training<br>• No measure of muscular strength
Corcos, D., J. Robichaud, F. David, D. Vaillancourt, C. Poon, M. Rafferty, C. Comella, W. Kohrt, and S. Leurgans, *24 months of exercise improves the motor symptoms in Parkinson's disease.* Neurology, 2012. **78**(1).	No full text available
Correa, C.L., A. De O.M. de Jesus, and V.L.S. De Britto, *Conventional physical therapy versus physical conditioning in patients with Parkinson's disease.* Movement Disorders, 2013. **28**: p. S150.	No full text available
Corcos, D.M., J.A. Robichaud, F.J. David, S.E. Leurgans, D.E. Vaillancourt, C. Poon, M.R. Rafferty, W.M. Kohrt, and C.L. Comella, *A two-year randomized controlled trial of progressive resistance exercise for Parkinson's disease.* Movement Disorders, 2013.	Both groups performed strengthening exercise
Cruise, K.E., R.S. Bucks, A.M. Loftus, R.U. Newton, R. Pegoraro, and M.G. Thomas, *Exercise and Parkinson's: benefits for cognition and quality of life.* Acta Neurologica Scandinavica, 2011. **123**(1): p. 13-9.	No measure of muscular strength

Study	Reason for exclusion
Curtis, C.L., C.C. Bassile, L.J. Cote, and A.M. Gentile, *Effects of exercise on the motor control of individuals with Parkinson's disease: case studies.* Neurology Report, 2001. **25**(1): p. 2-11.	No measure of muscular strength
Danoudis, M., M. Morris, J. McGinley, H. Menz, F. Huxham, J. Watts, A. Murphy, and R. Iansek, *Evaluation of movement strategy training in Parkinson's disease.* Movement Disorders, 2013. **28**: p. S156.	No full text available
Dashtipour K, Johnson E, Hadi E, White E, Ghamsary M, et al. (2014) Impact of exercise on the motor and non-motor symptoms of Parkinson's disease. Movement Disorders 29: S233.	No full text available
del Olmo, M.F., P. Arias, M.C. Furio, M.A. Pozo, and J. Cudeiro, *Evaluation of the effect of training using auditory stimulation on rhythmic movement in Parkinsonian patients--a combined motor and [18F]-FDG PET study.* Parkinsonism & Related Disorders, 2006. **12**(3): p. 155-64.	• No resistance training<br>• No measure of muscular strength
del Olmo, M.F. and J. Cudeiro, *Temporal variability of gait in Parkinson disease: effects of a rehabilitation programme based on rhythmic sound cues.* Parkinsonism & Related Disorders, 2005. **11**(1): p. 25-33.	No resistance training
Demonceau M, Rodrigues De La Cruz MC, Naveau F, Croisier JL, Maquet D, et al. (2013) Strength improvement after 3 months of resistance training among Parkinson s disease patients. Annals of Physical and Rehabilitation Medicine 56: e196.	No full text available
Dibble, L., T.F. Hale, J. Gerber, J. Droge, R.L. Marcus, and P.C. LaStayo, *The safety and feasibility of high intensity negative work in persons with Parkinson's disease.* Journal of Geriatric Physical Therapy, 2004. **27**(3): p. 117-117.	No full text available
Dibble, L.E., T. Hale, R.L. Marcus, J.P. Gerber, and P.C. LaStayo, *The Safety and Feasibility of High-Force Eccentric Resistance Exercise in Persons With Parkinson's Disease.* Archives of Physical Medicine & Rehabilitation, 2006. **87**(9): p. 1280-1282.	Not a parallel-group design (no control group)
Dibble, L.E., T.F. Hale, R.L. Marcus, J. Droge, J.P. Gerber, and P.C. LaStayo, *High-intensity resistance training amplifies muscle hypertrophy and functional gains in persons with Parkinson's disease.* Movement Disorders, 2006. **21**(9): p. 1444-52.	Both groups performed resistance training
Dibble, L.E., T.F. Hale, R.L. Marcus, J.P. Gerber, and P.C. LaStayo, High intensity eccentric resistance training decreases bradykinesia and improves Quality Of Life in persons with Parkinson's disease: a preliminary study. Parkinsonism & Related Disorders, 2009. 15(10): p. 752-7.	Both groups performed resistance training
Dibble, L.E., O. Addison, R.L. Marcus, K.B. Foreman, and P.C. LaStayo, *Skeletal muscle quality, muscle function, and mobility of persons with moderate Parkinson's disease improves in response to exercise.* Movement Disorders, 2012. **27**: p. S114.	No full text available

**Study**	**Reason for exclusion**
Diehl, D., S. Combs, and B. Staples, *Comparison of boxing training and traditional exercise on balance and quality of life outcomes in persons with Parkinson's disease: A pilot study.* Movement Disorders, 2011. **26**: p. S124.	No full text available
Difrancsico-Donoghue, J., W.G. Werner, and E.M. Lamberg, *The effects of exercise and B vitamins on glutathione levels via the transsulfuration pathway in Parkinsons disease.* Neurodegenerative Diseases, 2011. **8**.	No full text available
Dini, M., S. Corbianco, C. Ciappetta, P. Bongioanni, and B. Rossi, *Eccentric training for motor rehabilitation of parkinsonian patients.* European Journal of Neurology, 2009. **16**(S3): p. 308.	No full text available
Domingos, J.M.M. and J.J. Ferreira, *Feasibility study of an intensive exercise and educational program for Parkinson's disease.* Movement Disorders, 2012. **27**: p. S300-S301.	No full text available
D'Souza N, Anjali S, Sanghavi KP, Barretto M (2013) A study of the effect of a one year community-based group exercise program for people with Parkinson's disease in Mumbai, India: A quasi-experimental design. Journal of Parkinson's Disease 3: 142.	No outcome measure of strength
Ebersbach, G., A. Ebersbach, D. Edler, O. Kaufhold, M. Kusch, A. Kupsch, and J. Wissel, *Comparing exercise in Parkinson's disease--the Berlin LSVTBIG study.[Erratum appears in Mov Disord. 2010 Oct 30;25(14):2478].* Movement Disorders, 2010. **25**(12): p. 1902-8.	No measure of muscular strength
Ebersbach G, Ebersbach A, Gandor F, Wegner B, Wissel J, et al. (2014) Impact of physical exercise on reaction time in patients with parkinson's disease - Data from the berlin BIG study. Archives of physical medicine and rehabilitation. pp. 996-999.	No measure of muscular strength
Ehab, G., S. Barsnley, and R. Chellappa, *Effect of physical exercise-movement strategies programme on mobility, falls, and quality of life in Parkinson's disease.* International Journal of Therapy & Rehabilitation, 2012. **19**(2): p. 88-96.	No measure of muscular strength
Ellis, T., D.I. Katz, D.K. White, T.J. DePiero, A.D. Hohler, and M. Saint-Hilaire, *Effectiveness of an Inpatient Multidisciplinary Rehabilitation Program for People With Parkinson Disease.* Physical Therapy, 2008. **88**(7): p. 812-819.	No measure of muscular strength
Ellis, T.D., *Efficacy of rehabilitation in patients with Parkinson's disease*, 2005, Boston University. p. 127	No full text available
Fader, S.L., *Qi Gong exercise to improve balance for Parkinson fall prevention*, 2008, University of Massachusetts Amherst. p. 187	No full text available
Farley, B.G. and G.F. Koshland, *Training BIG to move faster: the application of the speed-amplitude relation as a rehabilitation strategy for people with Parkinson's disease.* Experimental Brain Research, 2005. **167**(3): p. 462-7.	• No measure of muscular strength<br>• No resistance training

Study	Reason for exclusion
Fisher, B.E., A.D. Wu, G.J. Salem, J. Song, C.H. Lin, J. Yip, S. Cen, J. Gordon, M. Jakowec, and G. Petzinger, *The effect of exercise training in improving motor performance and corticomotor excitability in people with early Parkinson's disease.* Archives of Physical Medicine & Rehabilitation, 2008. **89**(7): p. 1221-9.	No measure of muscular strength
Fletcher, E., V.A. Goodwin, S.H. Richards, J.L. Campbell, and R.S. Taylor, *An exercise intervention to prevent falls in Parkinson's: an economic evaluation.* BMC Health Services Research, 2012. **12**: p. 426.	No measure of muscular strength
Fletcher, E., V.A. Goodwin, S.H. Richards, J.L. Campbell, and R.S. Taylor, *An exercise intervention to prevent falls in Parkinson's disease: An economic evaluation.* Movement Disorders, 2013. **28**: p. S139.	• No full text available<br>• No outcome measure of strength
Foreman, K.B., O. Addison, R.L. Marcus, P.C. LaStayo, and L.E. Dibble, *Exercise and medication induced improvements in postural instability and dynamic balance task performance in persons with Parkinson's disease.* Movement Disorders, 2012. **27**: p. S114-S115.	No full text available
Formaggio, P.M., N.M. Rinaldi, C. Teixeira-Arroyo, M.P. Pereira, C.B. Takaki, V. Ralie, F. Stella, and L.T.B. Gobbi, *The effects of a multi-mode exercise program on Parkinson's disease postural control.* Movement Disorders, 2011. **26**: p. S127.	No full text available
Foster, E.R., L. Golden, R.P. Duncan, and G.M. Earhart, *Community-based Argentine tango dance program is associated with increased activity participation among individuals with Parkinson's disease.* Archives of Physical Medicine & Rehabilitation, 2013. **94**(2): p. 240-9.	No resistance training
Frazzitta, G., R. Maestri, G. Bertotti, D. Uccellini, G. Bazzini, P. Abelli, and R. Aquilani, *Rehabilitation in Parkinson's disease: assessing the outcome using objective metabolic measurements.* Movement Disorders, 2010. **25**(5): p. 609-14.	No resistance training
Frazzitta, G., G. Bertotti, M. Morelli, G. Riboldazzi, E. Pelosin, P. Balbi, N. Boveri, C. Comi, M. Turla, S. Leva, G. Felicetti, and R. Maestri, *Rehabilitation improves dyskinesias in Parkinsonian patients: a pilot study comparing two different rehabilitative treatments.* Neurorehabilitation, 2012. **30**(4): p. 295-301.	• No resistance training<br>• No measure of muscular strength
Frazzitta, G., M. Morelli, G. Bertotti, G. Felicetti, G. Pezzoli, and R. Maestri, *Intensive rehabilitation treatment in parkinsonian patients with dyskinesias: A preliminary study with 6-month followup.* Parkinson's Disease, 2012.	• No resistance training<br>• No measure of muscular strength
Frazzitta, G., G. Riboldazzi, G. Bertotti, M. Perini, D. Uccellini, G. Guaglio, M. Turla, C. Comi, G. Pezzoli, and R. Maestri, *Rasagilina and intensive rehabilitation: A randomized controlled study with 12 months follow-up.* Movement Disorders, 2012. **27**: p. S118-S119.	No full text available

Study	Reason for exclusion
Frazzitta G, Riboldazzi G, Bertotti G, Ferrazzoli D, Boveri N, et al. (2014) Multidisciplinary intensive rehabilitation treatment and rotigotine in the early stages of Parkinson's disease: A randomized controlled study. Movement Disorders 29: S239.	No full text available
Fritz, S., A. Merlo-Rains, E. Rivers, B. Brandenburg, J. Sweet, J. Donley, H. Mathews, S. deBode, and B.A. McClenaghan, *Feasibility of intensive mobility training to improve gait, balance, and mobility in persons with chronic neurological conditions: a case series.* Journal of Neurologic Physical Therapy, 2011. **35**(3): p. 141-7.	• No measure of muscular strength<br>• Case series of different neurological conditions: only 1 PD patient
Gauthier, L., S. Dalziel, and S. Gauthier, *The benefits of group occupational therapy for patients with Parkinson's disease.* American Journal of Occupational Therapy, 1987. **41**(6): p. 360-5.	• No resistance training<br>• No measure of muscular strength
Gauthier, L. and S. Gauthier, *Functional rehabilitation of patients with Parkinson's disease.* Physiotherapy Canada, 1983. **35**(4): p. 220-222.	Review/commentary
Gobbi, L.T., M.D. Oliveira-Ferreira, M.J. Caetano, E. Lirani-Silva, F.A. Barbieri, F. Stella, and S. Gobbi, *Exercise programs improve mobility and balance in people with Parkinson's disease.* Parkinsonism & Related Disorders, 2009. **15 Suppl 3**: p. S49-52.	No measure of muscular strength
Gobbi, L.T.B., R.A. Batistela, C. Teixeira-Arroyo, N.M. Rinaldi, E. Lirani-Silva, P.C.R. Santos, F.A. Barbieri, and R. Vitorio, *Dynamic balance improvement and executive function maintenance with a multimodal exercise program in Parkinson's disease.* Movement Disorders, 2011. **26**: p. S129.	No full text available
Gobbi, L.T.B., S. Gobbi, C. Teixeira-Arroyo, N.M. Rinaldi, F.A. Barbieri, E. Lirani-Silva, R.A. Batistela, M.P. Pereira, and F. Stella, *Eighteen months of intervention with exercise improve functional mobility with maintenance of mental state in people with Parkinson's disease.* Movement Disorders, 2010. **25**: p. S296-S297.	No full text available
Gobbi, L.T.B., M. P. Pereira, F. Stella, E. Lirani-Silva, P.C.R. Santos, C. Teixeira-Arroyo, N.M. Rinaldi, and R.A. Batistela, *Exercises programs affect balance, functional mobility and clinical parameters in Parkinson's disease according to its progression.* Movement Disorders, 2011. **26**: p. S129.	No full text available
Gobbi, L.T.B., R. Vitorio, C. Teixeira-Arroyo, E. Lirani-Silva, N.M. Rinaldi, F.A. Barbieri, M.P. Pereira, P.C.R. Santos, and R.A. Batistela, *Physical exercise in Parkinson's disease: Effects on gait velocity and attention.* Movement Disorders, 2012. **27**: p. S119-S120.	No full text available

Study	Reason for exclusion
Gobbi, R., L. Gobbi, M. Oliveira-Ferreira, A. Salles, C. Teixeira-Arroyo, N. Rinaldi, F. Stella, and S. Gobbi, *Effects of a multi-mode exercise program on quality of life and overall physical activity level in people with Parkinson's disease.* Parkinsonism and Related Disorders, 2009. **15**: p. S138.	No full text available
Goodwin, V., S. Richards, P. Ewings, A. Taylor, and J. Campbell, *Preventing falls in Parkinson's disease: The GETuP trial.* Parkinsonism and Related Disorders, 2009. **15**: p. S83.	No full text available
Goodwin, V.A., S.H. Richards, W. Henley, P. Ewings, A.H. Taylor, and J.L. Campbell, *An exercise intervention to prevent falls in people with Parkinson's disease: a pragmatic randomised controlled trial.* Journal of Neurology, Neurosurgery & Psychiatry, 2011. **82**(11): p. 1232-8.	No measure of muscular strength
Gruber, R.A. and J.H. Goldstein Elman, *Preliminary outcome evaluation of a modified LSVT BIG protocol for improved motor symptoms, physical mobility and quality of life (QOL) in people with Parkinson's disease (PD).* Movement Disorders, 2011. **26**: p. S292-S293.	No full text available
Guo, L., Y. Jiang, H. Yatsuya, Y. Yoshida, and J. Sakamoto, *Group education with personal rehabilitation for idiopathic Parkinson's disease.* Canadian Journal of Neurological Sciences, 2009. **36**(1): p. 51-9.	No measure of muscular strength
Hackney, M.E., *Argentine tango as therapy for Parkinson disease*, 2009, Washington University in St. Louis. p. 144 p.	No full text available
Hackney, M.E., S. Kantorovich, R. Levin, and G.M. Earhart, *Effects of tango on functional mobility in Parkinson's disease: a preliminary study.* Journal of Neurologic Physical Therapy, 2007. **31**(4): p. 173-9.	No measure of muscular strength
Harmer, P. and F. Li, *Self-report benefits of Tai Chi training by patients with Parkinson's disease.* Movement Disorders, 2013. **28**: p. S117.	No full text available
Hass, C.J., T.A. Buckley, C. Pitsikoulis, and E.J. Barthelemy, Progressive resistance training improves gait initiation in individuals with Parkinson's disease. Gait & Posture, 2012. 35(4): p. 669-73.	Control group did not perform 1RM strength test but only the biomechanical measurements. Thus, no comparison possible between intervention and control group for the outcome measure of strength.
Hass, C.J., M.A. Collins, and J.L. Juncos, Resistance training with creatine monohydrate improves upper-body strength in patients with Parkinson disease: a randomized trial. Neurorehabilitation & Neural Repair, 2007. 21(2): p. 107-15.	Both groups performed resistance training (one group with supplements, one group without)
Hughes, M.D., J.L. Trilk, R.B. Smith, and C.V. Skahen, *Feasibility and efficacy of a 16-week SpeedFlex exercise therapy program in patients with Parkinson's disease.* Movement Disorders, 2013. **28**: p. S172-S173.	No full text available

Study	Reason for exclusion
Hurwitz, A., *The benefit of a home exercise regimen for ambulatory Parkinson's disease patients.* Journal of Neuroscience Nursing, 1989. **21**(3): p. 180-4.	No measure of muscular strength
Jacobs, M., J. Fasano, M. Seyboth, E. Johnson, B. Marcoux, and A.M. Dupre, *The effect of an aquatic exercise program on balance in individuals with Parkinson Disease.* Journal of Aquatic Physical Therapy, 2012. **19**(2): p. 4-15.	No measure of muscular strength
Jaros, E. and D.J. Burn, *The role of sensory cues in the rehabilitation of Parkinsonian patients: A comparison of two physical therapy protocols.* Movement Disorders, 2000. **15**(5): p. 879-883.	• No resistance training<br>• No measure of muscular strength
Joudoux, S., T. Santiago, E. Hutin, N. Bayle, and J.M. Gracies, *Randomised controlled single-blind trial comparing two rehabilitation programs in Parkinson's disease at a moderate stage: Methodology.* Annals of Physical and Rehabilitation Medicine, 2011. **54**: p. e104.	No full text available
Ju-Hyun, K., L. Jeong-Uk, K. Mee-Young, K. Il-Hyun, K. Bokyung, and K. Junghwan, *The Effect of Standing Posture-enhancing Exercise on Parkinson's Disease Patients' Turning Around Motion.* Journal of Physical Therapy Science, 2012. **24**(10): p. 1047-1050.	No measure of muscular strength
Kara, B., A. Genc, B.D. Colakoglu, and R. Cakmur, *The effect of supervised exercises on static and dynamic balance in Parkinson's disease patients.* NeuroRehabilitation, 2012. **30**(4): p. 351-357.	No measure of muscular strength
Kargarfard, M., A. Chitsaz, and S. Azizi, *Effects of an 8-week aquatic exercise training on balance in patients with Parkinson's disease.* Journal of Isfahan Medical School, 2012. **30**(178).	No measure of muscular strength
Kelly NA, Ford MP, Standaert DG, Watts RL, Bickel CS, et al. (2014) Novel, high-intensity exercise prescription improves muscle mass, mitochondrial function, and physical capacity in individuals with Parkinson's disease. Journal of Applied Physiology 116: 582-592.	Control group was non-PD
Knobl, P.E. and Q.J. Almeida, *Rehabilitation for Parkinson's disease: Is it the sensory or attention focus that improves disease severity in sensory-attention focused interventions?* Movement Disorders, 2011. **26**: p. S153.	No full text available
Koc, A., *The effects of home exercise program on balance and activity' of daily living in parkinsonian patients.* Parkinsonism and Related Disorders, 2012. **18**: p. S153.	No full text available
Kwok, C.M., K.F. Hui, P.W. Ng, H.T. Lui, C.Y. Yick, K.Y. Wong, and T.K. Au, *Walking and low intensity strengthening program facilitate ambulatory, Motor and quality of life improvement in people with Parkinson's disease.* Parkinsonism and Related Disorders, 2012. **18**: p. S152.	No full text available

**Study**	**Reason for exclusion**
Lee, K.S., W.H. Lee, and S. Hwang, *Modified constraint-induced movement therapy improves fine and gross motor performance of the upper limb in Parkinson disease.* American Journal of Physical Medicine & Rehabilitation, 2011. **90**(5): p. 380-6.	No measure of muscular strength
Li, F. and K. Fitzgerald, *Postural stability in Parkinson's disease patients after Tai Chi training: A randomized controlled trial.* Parkinsonism and Related Disorders, 2012. **18**: p. S155.	No full text available
Lima, L.O. and F. Rodrigues-De-Paula, *Recruitment rate, feasibility and safety of power training in individuals with Parkinson's disease: a proof-of-concept study. / Taxa de recrutamento, viabilidade e segurança de um treinamento de potência muscular em indivíduos com doença de Parkinson: um estudo prova de conceito.* Brazilian Journal of Physical Therapy / Revista Brasileira de Fisioterapia, 2013. **17**(1): p. 49-56.	No outcome measure of muscular strength
Lindop, F.A., R.H. Skelly, and R. Smith, *Evaluation of an Otago-based exercise group for people with Parkinson's disease.* Movement Disorders, 2012. **27**: p. S307.	No full text available
Lomaglio MJ, Mallini KC (2013) Group exercise improves gait and balance in parkinson's disease but gains are lost over time. Archives of Physical Medicine and Rehabilitation 94: e12-e13.	No full text available
Loureiro, A.P.C., T.G. Gnoato, J.R. Viana, L. Cidade, J. Sabino, and L. Cruz, *Aquatic physical therapy approach, using principles of Halliwick Concept, for improvement of aquatic motor skills, among Parkinson's disease patients.* Parkinsonism and Related Disorders, 2009. **15**: p. S191.	No full text available
Lun, V., N. Pullan, N. Labelle, C. Adams, and O. Suchowersky, *Comparison of the effects of a self-supervised home exercise program with a physiotherapist-supervised exercise program on the motor symptoms of Parkinson's disease.* Movement Disorders, 2005. **20**(8): p. 971-5.	No measure of muscular strength
Mak, M.K. and C.W. Hui-Chan, Cued task-specific training is better than exercise in improving sit-to-stand in patients with Parkinson's disease: A randomized controlled trial. Movement Disorders, 2008. 23(4): p. 501-9.	No measure of muscular strength
Maki, T., E. Quagliato, and J.L. Duarte, *Effects of home exercises in patients with Parkinson's disease.* Parkinsonism and Related Disorders, 2009. **15**: p. S87.	No full text available
McGinley, J.L., C. Martin, F.E. Huxham, H.B. Menz, M. Danoudis, A.T. Murphy, J.J. Watts, R. Iansek, and M.E. Morris, *Feasibility, safety, and compliance in a randomized controlled trial of physical therapy for parkinson's disease.* Parkinson's Disease, 2012.	No measure of muscular strength

Study	Reason for exclusion
Meek, C., C.M. Sackley, C.E. Clarke, A.A. Soundy, C. Winward, P. Esser, S. Patel, and H. Dawes, *Long-term individual fitness enablement (LIFE) for Parkinson's disease: A feasibility study.* Movement Disorders, 2010. **25**: p. S713.	No full text available
Moore, C.G., M. Schenkman, W.M. Kohrt, A. Delitto, D.A. Hall, and D. Corcos, *Study in Parkinson Disease of Exercise (SPARX): Translating high-intensity exercise from animals to humans.* Contemporary Clinical Trials, 2013. **36**(1): p. 90-98.	• Study protocol only<br>• No measure of muscular strength<br>• No resistance training
Moriello, G., C. Denio, M. Abraham, D. DeFrancesco, and J. Townsley, *Incorporating yoga into an intense physical therapy program in someone with Parkinson's disease: A case report.* Journal of Bodywork and Movement Therapies, 2013.	No resistance training
Morris, M. and R. Iansek, *An interprofessional team approach to rehabilitation in Parkinson's disease.* European Journal of Physical Medicine & Rehabilitation, 1997. **7**(6): p. 166-170.	No resistance training
Morris, M.E., *Gait disorder and falls in Parkinson's disease: biomechanics and rehabilitation.* New Zealand Journal of Physiotherapy, 2009. **37**(3): p. 152-152.	No full text available
Morris, M.E., R. Iansek, and B. Kirkwood, *A randomized controlled trial of movement strategies compared with exercise for people with Parkinson's disease.* Movement Disorders, 2009. **24**(1): p. 64-71.	No measure of muscular strength
Morris, M.E., C. Martin, J.L. McGinley, F.E. Huxham, H.B. Menz, N.F. Taylor, M. Danoudis, J.J. Watts, S.E. Soh, A.H. Evans, M. Horne, and P. Kempster, *Protocol for a home-based integrated physical therapy program to reduce falls and improve mobility in people with Parkinson's disease.* BMC Neurology, 2012. **12**: p. 54.	• Study protocol only<br>• No measure of muscular strength
Morris, M.E., H. Menz, M. Danoudis, J. McGinley, R. Iansek, A. Murphy, F. Huxham, and J. Watts, *What are the best methods to prevent falls in Parkinson's disease?* Movement Disorders, 2013. **28**: p. S155.	No full text available
Morris, M.E., H.B. Menz, J.L. McGinley, F.E. Huxham, A.T. Murphy, R. Iansek, M. Danoudis, S.E. Soh, D. Kelly, and J.J. Watts, *Falls and mobility in Parkinson's disease: protocol for a randomised controlled clinical trial.* BMC Neurology, 2011. **11**: p. 93.	No measure of muscular strength
Munneke, M., M.J. Nijkrake, S.H. Keus, G. Kwakkel, H.W. Berendse, R.A. Roos, G.F. Borm, E.M. Adang, S. Overeem, B.R. Bloem, and G. ParkinsonNet Trial Study, *Efficacy of community-based physiotherapy networks for patients with Parkinson's disease: a cluster-randomised trial.* Lancet Neurology, 2010. **9**(1): p. 46-54.	No measure of muscular strength

Study	Reason for exclusion
Murtaugh, B., M.V. Albert, K. Karvelas, C.M. Marciniak, and S.D. Toledo, *Does a supervised program in physical therapy and/or occupational therapy improve pain scores in patients with parkinson disease?* PM and R, 2012. **4**(10): p. S314-S315.	No full text available
Myskja, A., *Evaluation of a rhythmic exercise program for patients with Parkinson's disease.* Movement Disorders, 2013. **28**: p. S106-S107.	No full text available
Nadeau, A., E. Pourcher, and P. Corbeil, *The effect of treadmill training on gait and quality of life in patients with early Parkinson's disease.* Movement Disorders, 2012. **27**: p. S309.	No full text available
Nascimento, C.M.C., C. Ayan, J.M. Cancela, L.T.B. Gobbi, S. Gobbi, and F. Stella, *Effect of a multimodal exercise program on sleep disturbances and instrumental activities of daily living performance on Parkinson's and Alzheimer's disease patients.* Geriatrics and Gerontology International, 2013.	No measure of muscular strength
Nica, A.S., G. Mologhianu, A. Murgu, C. Brailescu, L. Miron, B. Mitoiu, M. Ivascu, and T. Papacocea, *Possibilities and limits of rehabilitation program in Parkinson's disease and parkinsonian syndromes.* Parkinsonism and Related Disorders, 2012. **18**: p. S155.	No full text available
Nimwegen, M., A.D. Speelman, S. Overeem, B.P. Warrenburg, K. Smulders, M.L. Dontje, G.F. Borm, F.J. Backx, B.R. Bloem, and M. Munneke *Promotion of physical activity and fitness in sedentary patients with Parkinson's disease: randomised controlled trial*. BMJ (Clinical research ed.), 2013. **346**, f576.	No measure of muscular strength
Nocera, J., M. Horvat, and C.T. Ray, *Effects of home-based exercise on postural control and sensory organization in individuals with Parkinson disease.* Parkinsonism & Related Disorders, 2009. **15**(10): p. 742-5.	No measure of muscular strength
O'Brien, C.J., C.G. Canning, and L. Clemson, *Parkinson's disease: The experience of exercise.* Movement Disorders, 2013. **28**: p. S94.	No full text available
O'Brien, M., K.J. Dodd, and B. Bilney, *A qualitative analysis of a progressive resistance exercise programme for people with Parkinson's disease.* Disability & Rehabilitation, 2008. **30**(18): p. 1350-1357.	No measure of muscular strength
O'Callaghan AK, Jakovljevic DG, Trenell MI, Walker RW (2014) The effects of an exercise intervention on cardiovascular system and skeletal muscle function in idiopathic Parkinson's disease. Movement Disorders 29: S255.	No full text available
Palmer, S.S., J.A. Mortimer, D.D. Webster, R. Bistevins, and G.L. Dickinson, *Exercise therapy for Parkinson's disease.* Archives of Physical Medicine & Rehabilitation, 1986. **67**(10): p. 741-5.	No resistance training

Study	Reason for exclusion
Park A, Zid D, Russell J, Malone A, Rendon A, et al. (2014) Effects of a formal exercise program on Parkinson's disease: A pilot study using a delayed start design. Parkinsonism & related disorders. pp. 106-111.	No measure of muscular strength
Paul, S.S., C.G. Canning, J. Song, C. Sherrington, and V.S.C. Fung, *Leg muscle power training in Parkinson's disease: A randomised controlled trial.* Movement Disorders, 2013. **28**: p. S160.	No full text available
Paul SS, Canning CG, Song J, Fung VSC, Sherrington C (2014) Leg muscle power is enhanced by training in people with Parkinson’s disease: a randomized controlled trial. Clinical Rehabilitation 28: 275-288.	Both groups performed RT
Pedersen, S.W., B. Oberg, A. Insulander, and M. Vretman, Group training in parkinsonism: quantitative measurements of treatment. Scandinavian Journal of Rehabilitation Medicine, 1990. 22(4): p. 207-11.	Not a parallel-group design (no control group)
Peteet, J.O., *Self-management of Parkinson's disease: The effect of a group exercise program on lifestyle physical activity, self-efficacy, and function*, 2002, Walden University. p. 163 p.	No full text available
Peters, C., M. Currin, A. Rogers, S. Tyson, T. Comans, S. Healy, S. McPhail, K. Heathcote, and S. Brauer, *Effectiveness of an enhanced multidisciplinary community based group program for Parkinson's disease.* Movement Disorders, 2010. **25**: p. S716.	No full text available
Pickut, B., W. Van Hecke, E. Kerckhofs, P. Parizel, and P. Cras, *Mindfulness based training in parkinson's disease leads to increases in grey matter density.* Neurology, 2013. **80**(1).	No resistance training
Pickut, B.A., W. Van Hecke, E. Kerckhofs, D. Crosiers, P.M. Parizel, and P. Cras, *Gray matter density increases following a mindfulness based intervention in Parkinson's disease.* Movement Disorders, 2013. **28**: p. S41-S42.	No full text available
Pohl, M., G. Rockstroh, S. Ruckriem, G. Mrass, and J. Mehrholz, *Immediate effects of speed-dependent treadmill training on gait parameters in early Parkinson's disease.* Archives of Physical Medicine & Rehabilitation, 2003. **84**(12): p. 1760-6.	No resistance training
Poliakoff, E., A.J. Galpin, K. McDonald, M. Kellett, J.P. Dick, S. Hayes, and A.J. Wearden, *The effect of gym training on multiple outcomes in Parkinson's disease: a pilot randomised waiting-list controlled trial.* Neurorehabilitation, 2013. **32**(1): p. 125-34.	• No resistance training<br>• No measure of muscular strength
Pompeu, J.E., F.A. Mendes, K.G. Silva, A.M. Lobo, P. Oliveira Tde, A.P. Zomignani, and M.E. Piemonte, *Effect of Nintendo WiiTM-based motor and cognitive training on activities of daily living in patients with Parkinson's disease: a randomised clinical trial.* Physiotherapy, 2012. **98**(3): p. 196-204.	No measure of muscular strength

Study	Reason for exclusion
Pompeu, J.E., F.A. Mendes, K.G. Silva, A.M. Lobo, T.P. Oliveira, A.P. Zomignani, S.M.A.A. Pompeu, and M.E.P. Piemonte, *Functional improvement in patients with Parkinson's disease after balance and cognitive training in real or virtual environments.* Movement Disorders, 2012. **27**: p. S134-S135.	No full text available
Pompeu, J.E., F.A. Mendes, K.G. Silva, T.P. Oliveira, A.M. Lobo, S.M.A.A. Pompeu, A.P. Zomignani, and M.E.P. Piemonte, *Gait improvement in patients with Parkinson's disease after training in real and virtual environments.* Movement Disorders, 2012. **27**: p. S134.	No full text available
Pretzer-Aboff, I., E. Galik, and B. Resnick, *Feasibility and impact of a function focused care intervention for Parkinson's disease in the community.* Nursing Research, 2011. **60**(4): p. 276-83.	No measure of muscular strength
Pretzer-Aboff, I., B. Resnick, and E. Galik, *Long term impact of a restorative care intervention for people with Parkinson's in the community setting.* Movement Disorders, 2010. **25**: p. S682.	No full text available
Qutubuddin, A., T. Reis, R. Alramadhani, D.X. Cifu, A. Towne, and W. Carne, *Parkinson's disease and forced exercise: A preliminary study.* Rehabilitation Research and Practice, 2013. **2013**.	No resistance training
Qutubuddin, A.A., D.X. Cifu, P. Armistead-Jehle, W. Carne, T.E. McGuirk, and M.S. Baron, *A comparison of computerized dynamic posturography therapy to standard balance physical therapy in individuals with Parkinson's disease: a pilot study.* Neurorehabilitation, 2007. **22**(4): p. 261-5.	No resistance training
Rafferty, M.R., J.A. Robichaud, F.J. David, C. Poon, D.E. Vaillancourt, C.L. Comella, S. Leurgans, W.M. Kohrt, and D.M. Corcos, *Long-term exercise improves and maintains physical function in people with Parkinson's disease.* Movement Disorders, 2012. **27**: p. S135.	No full text available
Reuter, I., M. Engelhardt, K. Stecker, and H. Baas, Therapeutic value of exercise training in Parkinson's disease. Medicine & Science in Sports & Exercise, 1999. 31(11): p. 1544-9.	Not a parallel-group design (no control group)
Rinaldi, N.M., C. Teixeira-Arroyo, E. Lirani-Silva, P.H.S. Pelicioni, D.O. Silva, L. Simieli, F. Stella, and L.T.B. Gobbi, *Long-term physical exercise can improve cognitive function in people with Parkinson's disease.* Movement Disorders, 2011. **26**: p. S160-S161.	No full text available
Robichaud, J., K.D. Pfann, C.L. Comella, and D.M. Corcos, *Case study: exercise can improve muscle activation parameters and motor UPDRS in individuals with Parkinson's disease (PD)... Platforms, thematic posters, and posters for CSM 2007.* Journal of Neurologic Physical Therapy, 2006. **30**(4): p. 213-213.	No full text available

**Study**	**Reason for exclusion**
Robichaud, J.A., F.J. David, C. Poon, M. Rafferty, D.E. Vaillancourt, C.L. Comella, S. Leurgans, W.M. Kohrt, and D.M. Corcos, *Long-term progressive exercise improves bradykinesia and muscle weakness in Parkinson's disease.* Movement Disorders, 2012. **27**: p. S136.	No full text available
Rodrigues de Paula, F., L.F. Teixeira-Salmela, C.D. Coelho de Morais Faria, P. Rocha de Brito, and F. Cardoso, *Impact of an exercise program on physical, emotional, and social aspects of quality of life of individuals with Parkinson's disease.* Movement Disorders, 2006. **21**(8): p. 1073-7.	No measure of muscular strength
Rodriguez-Ruiz D, Guimaraes-Ribeiro D, Palomino A, Garcia-Manso JM, Martin-Gonzalez JM (2014) Effect of aquatic exercise program intervention on the complexity of the center of pressure oscillations time series in patients diagnosed with Parkinson's disease. Movement Disorders 29: S261-S262.	No full text available
Rodriguez-Ruiz D, Palomino A, Gutierrez S, Garcia D, Rodriguez-Matoso D (2014) Effects of different aquatic exercises programs on mechanical characteristics of vastus lateralis in subjects diagnosed with stage 1 Parkinson's disease. Movement Disorders 29: S262-S263.	No full text available
Sacheli, M.A. and Q.J. Almeida, *The addition of aerobic or resistance training to sensory attention focused exercise: An enhanced treatment for Parkinson's disease?* Movement Disorders, 2012. **27**: p. S136-S137.	No full text available
Sage, M.D. and Q.J. Almeida, *Symptom and gait changes after sensory attention focused exercise vs aerobic training in Parkinson's disease.* Movement Disorders, 2009. **24**(8): p. 1132-8.	• No resistance training<br>• No measure of muscular strength
Sage, M.D. and Q.J. Almeida, *A positive influence of vision on motor symptoms during sensory attention focused exercise for Parkinson's disease.* Movement Disorders, 2010. **25**(1): p. 64-9.	No measure of muscular strength
Sage, M.D. and Q.J. Almeida, *Exercise as a drug: A delayed start trial on the neuroprotective effects of exercise in Parkinson's disease.* Movement Disorders, 2010. **25**: p. S307-S308.	No full text available
Sage, M.D., R.E. Johnston, and Q.J. Almeida, *Comparison of exercise strategies for motor symptom improvement in Parkinsons disease.* Neurodegenerative Disease Management, 2011. **1**(5): p. 387-395.	No measure of muscular strength
Sakellari, V., G. Iatridou, A. Gializi, D. Pourotzoglou, G. Gioftsos, and V. Kiriakakis, *Therapeutic exercise and Parkinson's disease.* Review of Clinical Pharmacology and Pharmacokinetics, International Edition, 2005. **19**(2): p. 69-76.	No measure of muscular strength

Study	Reason for exclusion
Scandalis, T.A., A. Bosak, J.C. Berliner, L.L. Helman, and M.R. Wells, Resistance training and gait function in patients with Parkinson's disease. American Journal of Physical Medicine & Rehabilitation, 2001. 80(1): p. 38-43; quiz 44-6.	Not only participants with PD (one PD group performing RT, one healthy group performing RT)
Schalow, G., M. Paasuke, J. Ereline, and H. Gapeyeva, *Improvement in Parkinson's disease patients achieved by coordination dynamics therapy.* Electromyography & Clinical Neurophysiology, 2004. **44**(2): p. 67-73.	No resistance training
Schenkman, M., T.M. Cutson, M. Kuchibhatla, J. Chandler, C.F. Pieper, L. Ray, and K.C. Laub, *Exercise to improve spinal flexibility and function for people with Parkinson's disease: a randomized, controlled trial.* Journal of the American Geriatrics Society, 1998. **46**(10): p. 1207-16.	• No resistance training<br>• No measure of muscular strength
Schenkman, M., D.A. Hall, A.E. Baron, R.S. Schwartz, P. Mettler, and W.M. Kohrt, *Exercise for people in early- or mid-stage Parkinson disease: a 16-month randomized controlled trial.* Physical Therapy, 2012. **92**(11): p. 1395-410.	No measure of muscular strength
Shen, C.X. and M.K.Y. Mak, *Effects of 4-week compensatory step training on balance and gait performance in patients with Parkinson's disease.* Movement Disorders, 2010. **25**: p. S309.	No full text available
Shen, X. and M.K. Mak, *Repetitive step training with preparatory signals improves stability limits in patients with Parkinson's disease.* Journal of Rehabilitation Medicine, 2012. **44**(11): p. 944-9.	No measure of muscular strength
Shen, X. and M.K.Y. Mak, *Effects of 12-week strategy-focused balance training on balance performance and balance confidence in patients with Parkinson's disease.* Hong Kong Physiotherapy Journal, 2011. **29**(2): p. 100-100.	No full text available
Shen, X. and Y, *Effects of 8-week strategy-focused training on balance and gait performance in patients with Parkinson's disease... 7th Pan-Pacific Conference on Rehabilitation, Oct 23-24, 2010, Hong Kong.* Hong Kong Physiotherapy Journal, 2010. **28**: p. 26-26.	No full text available
Shulman, L.M., L.I. Katzel, F.M. Ivey, J. Sorkin, B. Smith, K. Anderson, T. Hill, J. Hammers, P.S. Fishman, B.J. Robottom, S.G. Reich, W.J. Weiner, and R.F. Macko, *Exercise and gait-related disability in parkinson disease.* Neurology, 2011. **77**(2): p. 198.	No full text available
Shulman, L.M., L.I. Katzel, F.M. Ivey, J.D. Sorkin, K. Favors, K.E. Anderson, S.G. Reich, W.J. Weiner, and R.F. Macko, *Does disease severity influence the efficacy of exercise in Parkinson's disease?* Movement Disorders, 2012. **27**: p. S139.	No full text available

Study	Reason for exclusion
Smania, N., E. Corato, M. Tinazzi, C. Stanzani, A. Fiaschi, P. Girardi, and M. Gandolfi, *Effect of balance training on postural instability in patients with idiopathic Parkinson's disease.* Neurorehabilitation & Neural Repair, 2010. **24**(9): p. 826-34.	• No resistance training<br>• No measure of muscular strength
Stack, E., H. Roberts, and A. Ashburn, *The PIT: SToPP trial-A feasibility randomised controlled trial of home-based physiotherapy for people with Parkinson's disease using video-based measures to preserve assessor blinding.* Parkinson's Disease, 2012.	No measure of muscular strength
States, R.A., D.K. Spierer, and Y. Salem, Long-term Group Exercise for People With Parkinson's Disease: A Feasibility Study. Journal of Neurologic Physical Therapy, 2011. 35(3): p. 122-128.	Not a parallel-group design (no control group)
Steffen, T., C. Petersen, and L. Dvorak, *Community-based exercise and wellness program for people diagnosed with Parkinson disease: experiences from a 10-month trial.* Journal of Geriatric Physical Therapy, 2012. **35**(4): p. 173-80.	No measure of muscular strength
Sung, H.R., B.K. Chae, H.Y. Jo, S.H. Choi, S.M. Cheon, and J.W. Kim, *Effect of the exercise program on postural stability in Parkinson's disease.* Movement Disorders, 2010. **25**: p. S293.	No full text available
Svircev, A., L.H. Craig, and J.L. Juncos *A pilot study examining the effects of neuromuscular therapy on patients with Parkinson's disease*. The Journal of the American Osteopathic Association, 2005. **105**, 26.	No resistance training
Tanaka, K., A.C. Quadros, Jr., R.F. Santos, F. Stella, L.T. Gobbi, and S. Gobbi, *Benefits of physical exercise on executive functions in older people with Parkinson's disease.* Brain & Cognition, 2009. **69**(2): p. 435-41.	No measure of muscular strength
Tanaka, K., R.F. Santos-Galduroz, F. Stella, L.T.B. Gobbi, and S. Gobbi, *Effect of physical exercise on visuospatial memory in older people with Parkinson's disease.* Neurodegenerative Diseases, 2011. **8**.	No full text available
Tickle-Degnen, L., T. Ellis, M.H. Saint-Hilaire, C.A. Thomas, and R.C. Wagenaar, *Self-management rehabilitation and health-related quality of life in Parkinson's disease: a randomized controlled trial.* Movement Disorders, 2010. **25**(2): p. 194-204.	No measure of muscular strength
Tsang, W.W.N., *Tai Chi training is effective in reducing balance impairments and falls in patients with Parkinson's disease.* Journal of Physiotherapy, 2013. **59**(1): p. 55-55.	Commentary only, not an intervention study
Uhlíř, P., J. Opavský, and A.M. Zaki Zaatar, *THE EFFECT OF REHABILITATION ON HEART RATE VARIABILITY IN PATIENTS WITH PARKINSON'S DISEASE. / EFEKT REHABILITACE NA VARIABILITU SRDEČNÍ FREKVENCE U PACIENTŮ S PARKINSONOVOU NEMOCÍ.* Acta Universitatis Palackianae Olomucensis. Gymnica, 2012. **42**(3): p. 49-54.	No resistance training<br>No measure of muscular strength

Study	Reason for exclusion
Vallabhajosula, S., J. Juncos, and C. Hass, *Effect of resistance training on sleep quality in persons with Parkinson disease.* Parkinsonism and Related Disorders, 2010. **16**: p. S61.	No full text available
Vitale, C., V. Agosti, D. Avella, G. Santangelo, M. Amboni, R. Rucco, P. Barone, F. Corato, and G. Sorrentino, *Effect of Global Postural Rehabilitation program on spatiotemporal gait parameters of parkinsonian patients: a three-dimensional motion analysis study.* Neurological Sciences, 2012. **33**(6): p. 1337-43.	No measure of muscular strength
Vivas, J., P. Arias, and J. Cudeiro, *Aquatic therapy versus conventional land-based therapy for Parkinson's disease: an open-label pilot study.* Archives of Physical Medicine & Rehabilitation, 2011. **92**(8): p. 1202-10.	No measure of muscular strength
Wade, D.T., H. Gage, C. Owen, P. Trend, C. Grossmith, and J. Kaye, *Multidisciplinary rehabilitation for people with Parkinson's disease: a randomised controlled study.* Journal of Neurology, Neurosurgery & Psychiatry, 2003. **74**(2): p. 158-62.	No measure of muscular strength
White, D.K., R.C. Wagenaar, T.D. Ellis, and L. Tickle-Degnen, *Changes in walking activity and endurance following rehabilitation for people with Parkinson disease.* Archives of Physical Medicine & Rehabilitation, 2009. **90**(1): p. 43-50.	No measure of muscular strength
Winward, C., C. Sackley, C. Meek, H. Izadi, K. Barker, D. Wade, and H. Dawes, *Weekly exercise does not improve fatigue levels in Parkinson's disease.* Movement Disorders, 2012. **27**(1): p. 143-6.	No measure of muscular strength
Yang, Y.R., Y.Y. Lee, S.J. Cheng, and R.Y. Wang, *Downhill walking training in individuals with Parkinson's disease: a randomized controlled trial.* American Journal of Physical Medicine & Rehabilitation, 2010. **89**(9): p. 706-14.	No measure of muscular strength
Yang, Y.R., Y.Y. Lee, S.J. Cheng, and R.Y. Wang, Downhill walking training in individuals with Parkinson's disease: a randomized controlled trial. American Journal of Physical Medicine & Rehabilitation, 2010. 89(9): p. 706-14.	No resistance training
Yousefi, B., V. Tadibi, A.F. Khoei, and A. Montazeri, *Exercise therapy, quality of life, and activities of daily living in patients with Parkinson disease: a small scale quasi-randomised trial.* Trials [Electronic Resource], 2009. **10**: p. 67.	No measure of muscular strength
